# Myosin binding protein H-like (MYBPHL): a promising biomarker to predict atrial damage

**DOI:** 10.1038/s41598-019-46123-w

**Published:** 2019-07-10

**Authors:** Harald Lahm, Martina Dreßen, Nicole Beck, Stefanie Doppler, Marcus-André Deutsch, Shunsuke Matsushima, Irina Neb, Karl Christian König, Konstantinos Sideris, Stefanie Voss, Lena Eschenbach, Nazan Puluca, Isabel Deisenhofer, Sophia Doll, Stefan Holdenrieder, Matthias Mann, Rüdiger Lange, Markus Krane

**Affiliations:** 1 Department of Cardiovascular Surgery, Division of Experimental Surgery, Institute Insure (Institute for Translational Cardiac Surgery), German Heart Center Munich at the Technical University of Munich, Munich, Germany; 2 Department of Thoracic and Cardiovascular Surgery, Herz- und Diabeteszentrum NRW, University Hospital Ruhr-University Bochum, Bad Oeynhausen, Germany; 3 Department of Cardiovascular Disease, German Heart Center Munich at the Technical University of Munich, Munich, Germany; 4 Department of Proteomics and Signal Transduction, Max Planck Institute of Biochemistry, Martinsried, Germany; 5 Institute of Laboratory Medicine, German Heart Center Munich, Munich, Germany; 6 Novo Nordisk Foundation Center for Protein Research, Faculty of Health Sciences, University of Copenhagen, Copenhagen, Denmark; 7 DZHK (German Center for Cardiovascular Research), Partner Site Munich Heart Alliance, Munich, Germany

**Keywords:** Predictive markers, Biomarkers

## Abstract

Myosin binding protein H-like (MYBPHL) is a protein associated with myofilament structures in atrial tissue. The protein exists in two isoforms that share an identical amino acid sequence except for a deletion of 23 amino acids in isoform 2. In this study, *MYBPHL* was found to be expressed preferentially in atrial tissue. The expression of isoform 2 was almost exclusively restricted to the atria and barely detectable in the ventricle, *arteria mammaria interna*, and skeletal muscle. After atrial damage induced by cryo- or radiofrequency ablation, MYBPHL was rapidly and specifically released into the peripheral circulation in a time-dependent manner. The plasma MYBPHL concentration remained substantially elevated up to 24 hours after the arrival of patients at the intensive care unit. In addition, the recorded MYBPHL values were strongly correlated with those of the established biomarker CK-MB. In contrast, an increase in MYBPHL levels was not evident in patients undergoing aortic valve replacement or transcatheter aortic valve implantation. In these patients, the values remained virtually constant and never exceeded the concentration in the plasma of healthy controls. Our findings suggest that MYBPHL can be used as a precise and reliable biomarker to specifically predict atrial myocardial damage.

## Introduction

The term clinical biomarker is used for any biological substance whose presence and detection support the decisions involving subsequent medical treatment or therapy. Its detection should be rapid, easy, reproducible, and relatively cheap. A biomarker for myocardial damage (e.g., myocardial infarction or ablation) should be exclusively expressed in the myocardium and should not be detectable in the peripheral circulation under normal physiological conditions. Furthermore, its rapid release into the blood at the time of injury and long-lasting detection are necessary for an adequate diagnostic window.

In cardiovascular research, the use of biomarkers dates back to the fifties of the last century when aspartate aminotransferase, lactate dehydrogenase, and creatine kinase were first introduced as markers for acute coronary syndrome^1–3^. Since then, the development of more reliable and highly sensitive biomarkers for acute myocardial infarction has led to the use of the cardiac troponins^4–7^.

In our previous work, we analyzed the proteome of sixteen different regions of the healthy human heart^8^. In this study, we defined a subset of proteins that showed a significantly higher expression in the atria compared to the ventricles. Myosin binding protein H-like (MYBPHL) showed the strongest tissue specificity. To date, very little is known about the biological role of MYBPHL, potential protein-protein interactions, or its exact subcellular localization. Recently, Barefield and colleagues showed that MYBPHL localizes to the myofilament in atrial tissue and may be associated with arrhythmia and dilated cardiomyopathy^9^. In addition, *mybphl* knock-out mice have abnormalities in the heart conduction system and increased arrhythmia^9^.

Here we show that *MYBPHL* is tremendously over-expressed in human atrial tissue compared to the ventricle or other non-cardiac tissues. In particular, the mRNA of *MYBPHL* isoform 2 is exclusively found in the atria. Furthermore, we provide evidence that an induced atrial damage by cryo- or radiofrequency ablation procedures in patients suffering from atrial fibrillation provokes a rapid, time-dependent and long-lasting release of MYBPHL into the plasma. In different control groups, no elevated levels of MYBPHL were detected. Thus, our data suggest that MYBPHL can serve as a potential new biomarker to assess cardiac injury, especially atrial damage.

## Results

### MYBPHL is selectively expressed in the atria of the human heart

**Figure 1:**
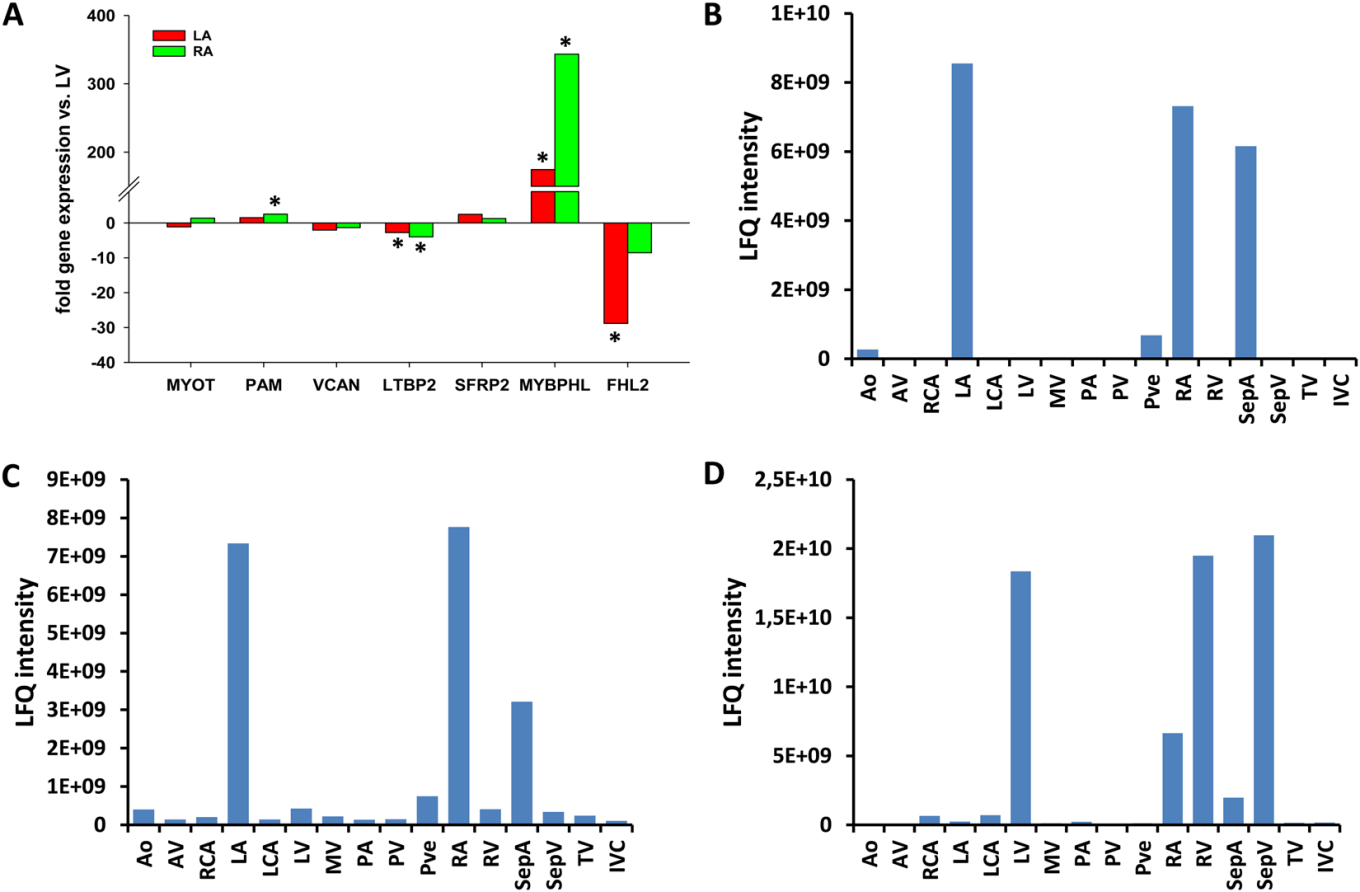
Expression of candidate genes in different tissues of the human heart. (**A**) Expression of candidate genes in left and right atrium (*n* = 3 each). Values represent the fold-change of gene expression compared to left ventricle. (**B**–**D**) Determination of the relative protein amount indicated as label-free quantification intensity of MYBPHL (**B**), PAM (**C**) and FHL2 (**D**) in 16 different regions of the human heart. Ao: aorta, AV: aortic valve, RCA: right coronary artery, LA: left atrium, LCA: left coronary artery, LV: left ventricle, MV: mitral valve, PA: pulmonary artery, PV: pulmonary valve, Pve: pulmonary vein, RA: right atrium, RV: right ventricle, SepA: atrial septum, SepV: ventricular septum, TV: tricuspid valve, IVC: inferior vena cava.

We previously analyzed the proteome of 16 different regions of the healthy human heart. In a comparison of the atrial and ventricular proteomes, we identified 1,220 proteins that were expressed significantly higher in the atria, and 409 proteins had higher expression in the ventricles^8^. To verify the differential gene expression between both locations in the human heart, we compared the expression of several candidate genes in left and right atrial tissue (LA and RA, respectively) to that in the left ventricle (LV) (Fig. 1A). We found significantly higher *MYBPHL* expression in both the LA (p = 0.032) and RA (p = 0.023) compared to the LV. In contrast, *four and a half LIM domains 2* (*FHL2*) showed strong expression in the LV but was barely detected in either atrium. Furthermore, *latent transforming growth factor beta binding protein 2* (*LTBP2*) was found at significantly higher levels in the LV (p = 0.044) whereas *peptidylglycine alpha-amidating monooxygenase* (*PAM*) was expressed at significantly higher levels in the RA (p = 0.044) compared to the LV. No apparent differences were observed for *myotilin* (*MYOT*), *versican* (*VCAN*), or *secreted frizzled-related protein 2* (*SFRP2*) between these areas of the heart.

After confirming differential gene expression in atrial and ventricular tissue, we analyzed the relative protein expression of these candidate genes in 16 different heart regions based on our previously published data^8^. MYBPHL expression was restricted to the LA, RA, and atrial septum but was virtually absent from all other tissues analyzed (Fig. 1B). PAM was also present in increased amounts in both atria and the atrial septum with low expression levels in all other cardiac regions (Fig. 1C). Of the other potential atrium-specific candidate genes, MYOT was limited to the left atrium and atrial septum while the expression of VCAN, LTBP2, and SFRP2 did not display a region-specific pattern (Supplementary Fig. S1). In contrast, FHL2 protein expression was almost exclusively restricted to both ventricles and the ventricular septum (Fig. 1D). Thus, our results suggest MYBPHL and FHL2 may be useful biomarkers for the atrial and ventricular heart regions, respectively.

**Figure 2:**
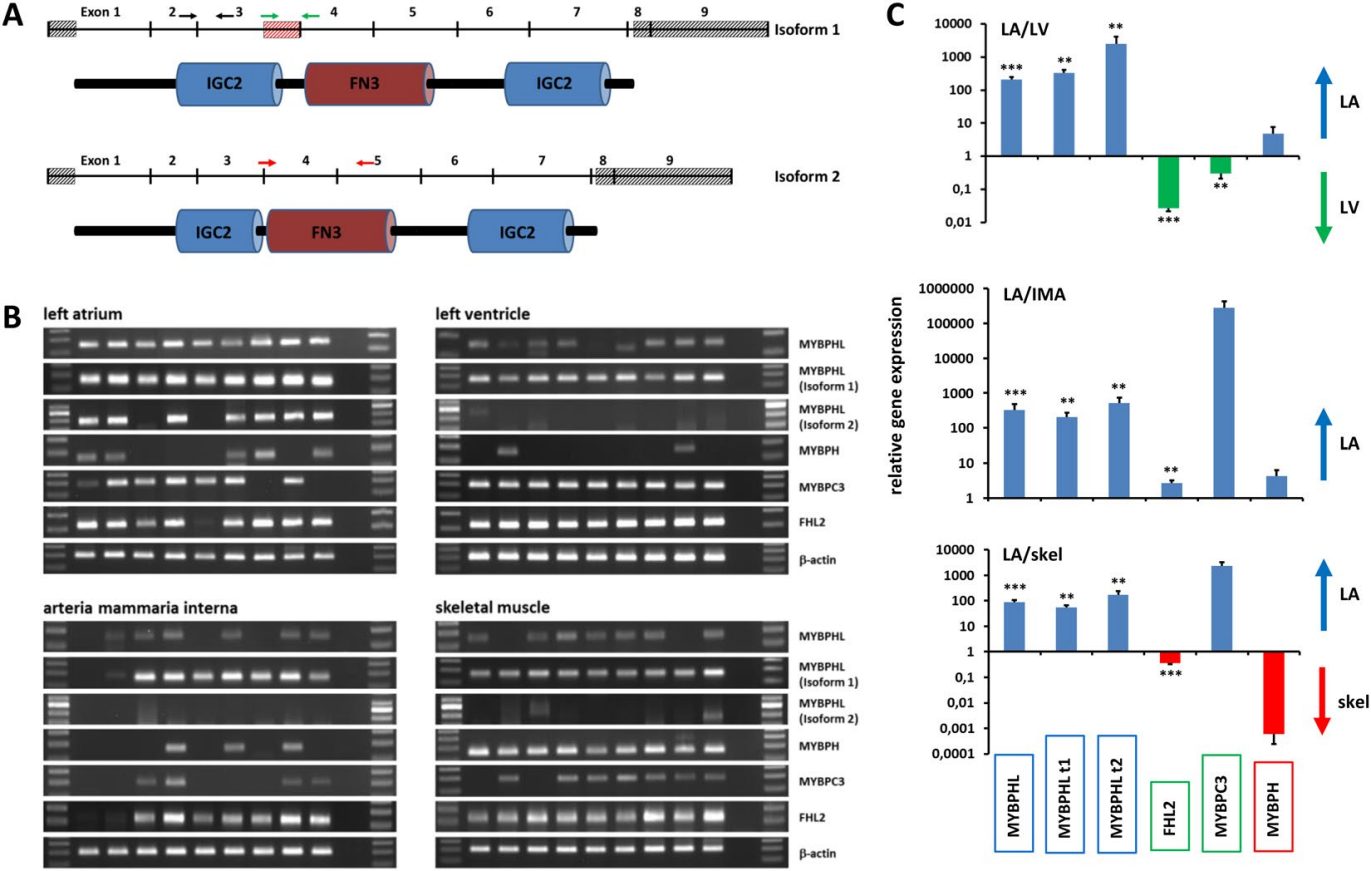
MYPHL isoform 2 is specifically expressed in human atrial tissue. (**A**) Structure of *MYBPHL* isoforms 1 and 2.  non-coding regions,  deleted in isoform 2. Arrows indicate the location of primers used to amplify *MYBPHL* (black arrows), isoform 1 (green arrows) or isoform 2 (red arrows). (**B**) Gene expression in human LA, LV, *arteria mammaria interna* and skeletal muscle tissue. In each slot of the gel 20 µL of amplified fragments were loaded. (**C**) Quantification of relative gene expression in LA vs. LV, *arteria mammaria interna* and skeletal muscle tissue. Values are expressed as the mean ± SE. *p < 0.05, **p < 0.01, ***p < 0.001.

*MYBPHL* is expressed as two different isoforms, which have an identical amino acid sequence except for a deletion of 23 amino acids in isoform 2 at positions 121 through 143 (Fig. 2A). We analyzed the tissue-specific expression of both *MYBPHL* isoforms, *MYBPH*, *MYBPC3*, and *FHL2* in cardiac tissue (LA and LV), arterial tissue (*arteria mammaria interna*), and skeletal muscle (Fig. 2B). *MYBPHL* transcripts were present in all four tissues but at a significantly higher level in the LA (p = 0.00045) (Fig. 2C). Using isoform-specific primers, we investigated whether the *MYBPHL* isoforms might display tissue-specific differential expression. *MYBPHL* isoform 1 transcripts were found throughout all four tissues analyzed, comparable to the results obtained for *MYBPHL*, and most abundant in the LA. In contrast, the *MYBPHL* isoform 2 was selectively and strongly expressed in 7 of 9 LA samples (Fig. 2B). Its expression level in the LA was 160- to 2,500-fold higher than in the LV, *arteria mammaria interna*, and skeletal muscle with absent or very low-level expression (Fig. 2B,C). In addition, we compared the expression of the paralog gene *MYBPH* and that of *MYBPC3*, which is closely related to *MYBPHL*. *MYBPH* transcripts were found abundantly in all skeletal muscle samples compared to cardiac muscle (LA and LV) and arterial tissue (Fig. 2B,C). *MYBPC3* was expressed in all four tissues, but predominantly in the LV (Fig. 2B,C). Finally, we analyzed the expression of *FHL2*, which was detected in all four tissues (Fig. 2B) with the highest expression in the LV (Fig. 2B,C). These data were consistent with that of our initial screen (Fig. 1A,D). Our results demonstrated that *MYBPHL* and its isoforms were predominantly expressed in atria, *MYBPC3* and *FHL2* in the ventricles, and *MYBPH* in skeletal muscle.

### MYBPHL concentration is increased in plasma after atrial myocardial injury and correlates with known biomarkers

Having established the specific and abundant expression of *MYBPHL* in atrial tissue, we speculated that atrial damage might be reflected in the blood of such patients. Therefore, we analyzed MYBPHL protein concentration in the plasma from 17 patients undergoing surgical cryo- or radiofrequency ablation for atrial fibrillation (Supplementary Table S1). The cryoablation was performed following a standardized protocol with ablation lines only in the left and right atrium. Therefore, these patients underwent a selectively induced atrial cardiac injury. These provoked atrial cardiac injuries are usually accompanied by strong elevation of known biomarkers (e.g., creatine kinase MB (CK-MB) and troponins). CK-MB and troponin levels are robustly increased post-operatively but do not differentiate between atrial cardiac damage due to cryoablation or a perioperative myocardial infarction. Therefore, a selective and specific biomarker is highly desirable for postoperative monitoring and decision-making for further invasive diagnostics (e.g., cardiac catheterization).

**Figure 3:**
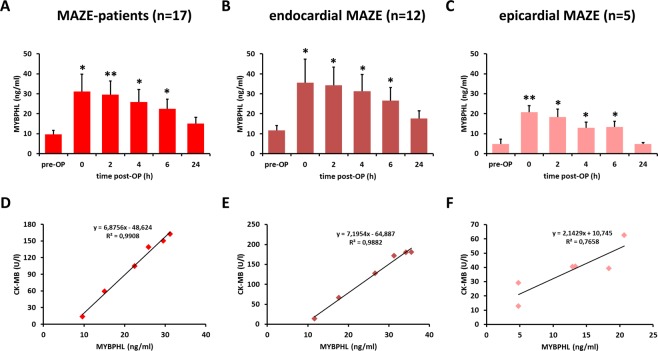
MYBPHL protein in plasma is increased after atrial damage. (**A**) Time course of MYBPHL in patients with atrial fibrillation receiving cryo-ablation (*n* = 17). (**B**,**C**) Time course of MYBPHL expression in atrial fibrillation patients with endo- (**B**) (*n* = 12) or epicardial (**C**) (*n* = 5) cryo-ablation. (**D**–**F**) Correlation between MYBPHL and CK-MB. Values are presented as the mean ± SEM. *p < 0.05, **p < 0.01.

MYBPHL plasma concentrations were measured pre-operatively, upon arrival at the intensive care unit (ICU), and 2, 4, 6 and 24 hours thereafter. We observed a more than 3-fold (9.66 ± 1.66 vs. 31.19 ± 8.56 ng/mL) increase in MYBPHL concentrations upon arrival at the ICU. Subsequently, the concentration gradually declined until 24 hours post-operation. The levels at this time point (15.11 ± 3.07 ng/mL) were still higher than pre-operative levels (Fig. 3A).

Next, we analyzed two different subgroups of ablation therapy. One group underwent endocardial cryoablation after the opening of the right and left atrium by performing a subset of at least six ablation lines, including box-lesions. These box-lesions created a circular isolation of both left and right pulmonary veins. Further lines connecting both box-lesions and additional lines were created to the left appendage and the mitral valve annulus^10^. The endocardial cryoablation was performed in patients undergoing mitral and/or tricuspid valve surgery. The second group underwent epicardial radiofrequency ablation of the pulmonary veins (Supplementary Table S1) concomitant with aortic valve replacement or coronary artery bypass grafting. In patients with endocardial cryoablation, MYBPHL values increased more than 3-fold to approximately 35 ng/mL upon arrival at the ICU (Fig. 3B). In contrast, MYBPHL levels only increased to 20 ng/mL in patients with epicardial ablation (Fig. 3C). This difference could be due to the more aggressive and comprehensive nature of endocardial cryoablation therapy compared to localized epicardial radiofrequency ablation. Thus, MYBPHL values were in good agreement with the degree of atrial damage. It is known that cardiac injury induced by ablation therapy also leads to elevated plasma levels of CK-MB and troponin T (markers of cardiac damage) released from atrial tissue (cardiomyocytes). In the current study, we also found significantly increased levels of both biomarkers over time (p < 0.001) (Supplementary Figs S2 and S3). Furthermore, the lower degree of atrial damage induced by radiofrequency ablation was also reflected by lower CK-MB values within this group (Supplementary Fig. S2).

Finally, we analyzed the correlation between MYBPHL and CK-MB concentrations at different time points. We obtained a good correlation for all MAZE patients and those who received either endocardial or epicardial ablation (R^2^ = 0.9908, 0.9882, and 0.7658, respectively) (Fig. 3D–F). In contrast, there was no correlation between the concentrations of MYBPHL and troponin T in these groups of patients (R^2^ between 0.0675 and 0.1867) (Supplementary Fig. S3). Thus, MYBPHL is a newly identified biomarker of cardiac damage that can rapidly detect cardiac necrosis upon arrival at the ICU. Furthermore, it is substantially elevated up to 24 hours after the procedure and correlates nicely with a known and established biomarker for cardiac damage (CK-MB).

### MYBPHL concentration in plasma of patients undergoing cardiac surgery without atrial myocardial injury

**Figure 4:**
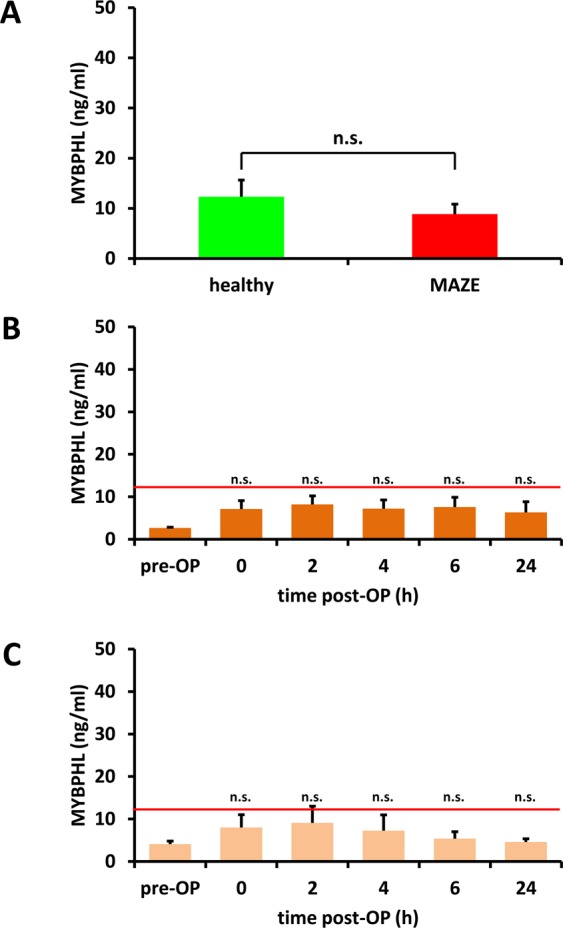
MYBPHL is not increased in plasma of patients without atrial damage. (**A**) Comparison of MYBPHL concentration in plasma of healthy volunteers (*n* = 11) with pre-OP values of patients with myocardial injury (*n* = 17). (**B**,**C**) Time course of MYBPHL in patients with AVR (**B**) (*n* = 5) or TAVI (**C**) (*n* = 5). The red line represents the mean concentration of MYBPHL in plasma of healthy controls. Values are presented as the mean ± SEM. n.s.: not significant.

To judge pre-operatively measured values of patients undergoing a MAZE procedure, we measured the concentration of MYBPHL in the plasma of 11 healthy volunteers. There was no significant difference (p = 0.368) in the MYBPHL concentrations of these healthy volunteers and the pre-operative levels of patients undergoing the MAZE procedure (Fig. 4A).

To further investigate the hypothesis that MYBPHL release into circulation could be used as a specific biomarker for myocardial injury, we analyzed two additional groups of surgical patients. The first group underwent aortic valve replacement (AVR) using a conventional surgical procedure with median sternotomy and cardiopulmonary bypass (Supplementary Table S2). During the whole period, the plasma MYBPHL concentrations did not deviate significantly from the pre-operative values for this group. In addition, the values did not exceed the concentrations measured in healthy volunteers at any of the time points evaluated (Fig. 4B). The second group underwent a transcatheter aortic valve implantation (TAVI). These patients routinely present with a higher age and more concomitant disease (Supplementary Table S2). The TAVI procedure does not need sternotomy, cardiopulmonary bypass, or cardioplegic arrest and is, therefore, a less invasive procedure compared to AVR. We found that MYBPHL concentrations in this patient group also remained similar to the pre-operative concentrations and never exceeded the levels in the plasma of healthy volunteers, except for a minor increase (p > 0.1) at the time of arrival at the ICU and two hours later (Fig. 4C). Furthermore, the MYBPHL concentrations measured in TAVI and AVR patients never reached the levels found in patients undergoing artificially induced atrial damage either by endo- or epicardial ablation (see Fig. 3).

Thus, our data strongly suggest that serum MYBPHL concentrations are only elevated in the case of myocardial damage. Furthermore, serum MYBPHL levels are similar between healthy volunteers and pre-operative values from patients suffering from cardiovascular disease.

### MYBPHL concentration in patients with atrioventricular node ablation

Recently, Barefield *et al*. described – beside the atrial expression – specific expression of MYBPHL in the cells of the conduction system of the heart^9^. Therefore, we aimed to quantify the release of MYBPHL in patient plasma after atrioventricular node (AVN) ablation (Supplementary Table S3). To this end, samples from a different study using a slightly differing protocol for blood sample withdrawal (Supplementary Fig. S4A) were analyzed for the plasma MYBPHL concentration. Pre-operative MYBPHL levels were higher in patients undergoing AVN ablation compared to all other groups, but the differences did not reach significance (p > 0.081). Furthermore, no significant differences in the plasma MYBPHL concentrations were observed between the pre- and post-procedural time points (up to 24 h post-procedure) (Supplementary Fig. S4B).

## Discussion

In our present work, we identified *MYBPHL* as a gene that is preferentially and most abundantly (up to 300-fold) expressed in human atria compared to the ventricles, *arteria mammaria interna*, or skeletal muscle. These results are similar to those of comparative microarray analyses^11,12^. In addition, these results are consistent with our previous work, which demonstrated that MYBPHL protein was strongly enriched in the atrial tissue of the human heart^8^. Additional examination of the distribution of MYBPHL protein in 16 cardiac tissues confirmed that it was absent or barely detectable in all but atrial tissue in the heart. Though *MYBPHL* is processed into two isoforms, their tissue specificity had not been previously evaluated. We detected isoform 1 transcripts in all four tissues examined. However, it was expressed predominantly in the left atrial tissue. With the exception of atrial tissue, isoform 2 transcripts were only detectable at negligible levels in a few samples. The closely related *MYBPC3* gene, which shares a fibronectin-3 and an immunoglobulin C-2 type domain at the C terminus with *MYBPHL*, was predominantly expressed in ventricular tissue while *MYBPH*, a paralog of *MYBPHL*, appeared to be a marker of skeletal muscle. Therefore, *MYBPHL* and, in particular, isoform 2 appeared to be expressed very selectively in the atria of the human heart, suggesting its potential use as an atrium-specific biomarker.

Currently, there are no existing biomarkers that can be used to specifically monitor damage to atrial tissue. Different cardiac-specific biomarkers (e.g., CK-MB, troponin T, and troponin I) have been evaluated following surgical- or catheter-based ablation procedures. All of these markers are significantly increased after ablation^13,14^. However, none of these have significant prognostic value, most probably due to their lack of specificity for the atrial myocardium. Nevertheless, monitoring the extent of atrial damage could be of paramount interest, especially after surgical- or catheter-based ablation procedures. A potential marker could indicate the short- or long-term success of a procedure by reflecting the specific degree of ablated atrial myocardium. Furthermore, an atrium-specific biomarker could be a valuable tool to differentiate between atrial and ventricular damage. Iatrogenic ventricular damage could occur, for example, during concomitant surgical procedures, such as mitral valve surgery by occlusion of a coronary vessel. Our results clearly show that MYBPHL can serve as a marker to detect such an injury. After cryo- or radiofrequency ablation, the concentration of MYBPHL in the plasma of these patients increased substantially after the operation and then gradually declined to almost pre-operative values 24 hours after the procedure. After the more intensive endocardial ablation, MYBPHL increased to higher values compared to the less extensive epicardial therapy. These results were in good agreement with the hypothesis that more severe damage in the atrium would induce a greater release of MYBPHL into the peripheral circulation.

To evaluate cardiac injury, highly sensitive assays have been established that measure the release of cardiac troponin T and I, especially upon acute myocardial infarction^7,15^. However, the increase in troponin can also be the result of conditions other than myocardial infarction (e.g., myocarditis and non-cardiac pathology, such as renal failure)^16,17^. To confirm the specificity of MYBPHL increase after atrial damage, we included additional control groups in our study. Firstly, we analyzed the time-course in patients with AVR, who needed the support of a heart-lung machine during the operation. Secondly, we investigated TAVI patients, who underwent a minimally invasive intervention. In both groups, the plasma concentration of MYBPHL did not change significantly from pre-operative levels and never went above the concentrations in the plasma of healthy individuals. Thus, our data demonstrate that MYBPHL plasma concentrations increase specifically after an atrial injury. Given the fact that *MYBPHL* isoform 2 has an expression pattern that is almost exclusively restricted to the atria, it would be most desirable to use discriminating antibodies against this isoform to further enhance the specificity. However, such an antibody is not currently available.

It is most desirable to detect any potential cardiac damage as early as possible. The time to obtain a positive and meaningful result after cardiac damage for the troponins or CK-MB levels ranges from one to three hours^7,18^. Our data demonstrated that *MYBPC3*, also known as cMyBP-C, is predominantly expressed in ventricular tissue. In a pig model, cMyBP-C was released quite early after ligation of the left anterior descending coronary artery (LAD), which induced a sizeable infarction. At this early time point (30 minutes after LAD ligation), the established biomarkers for myocardial infarction (cardiac troponin I and T) still remained at basal levels^19^. This time point is comparable to the period between the release of cross-clamping and start of reperfusion after ablation in patients in whom a modified MAZE procedure has been performed and their arrival at the ICU (60–90 min). Even after this short period, MYBPHL concentrations in the plasma were significantly increased over the pre-operative values. MYBPHL is closely related to MYBPC3 structurally. The latter is quite susceptible to proteolysis upon cardiac stress^20^. Due to their structural relationship, this proteolytic susceptibility may also be the case for MYBPHL and contribute to its early release into the periphery. The concentration of MYBPHL remained markedly elevated up to twenty-four hours post-operation. Therefore, plasma MYBPHL appears to be a biomarker for atrial damage that can be measured rather early and continuously for up to twenty-four hours.

The understanding of the potential role of MYBPHL in cardiac disease is very limited. The *MYBPHL* gene is located on human chromosome 1p13.3. This locus has been identified as one of the most significant risk factors for coronary artery disease (CAD)^21,22^. In addition, the same chromosomal locus is strongly associated with the concentration of LDL cholesterol^23,24^, a causal risk factor for CAD. MYBPHL is present in the myofilament fraction of the atria, which is abolished in a truncated form harboring a premature stop codon. This mutation has been discovered in a family where carriers suffered from cardiomyopathy and conduction system abnormalities^9^. The reported expression of MYBPHL in conduction system cells prompted us to measure MYBPHL concentration in patients undergoing selective AVN ablation. However, we were not able to show a significant increase in the MYBPHL concentration in the plasma over 24 hours in this patient population, which could be due to the low release of MYBPHL in the circulation by selective AVN ablation that causes only a small degree of damage. In addition, higher pre-procedural levels in this patient population may mask significantly increased levels due to the ablation.

In conclusion, we have identified *MYBPHL* as a gene that is predominantly expressed in atrial tissue, especially *MYBPHL* isoform 2, which is barely detectable in the ventricles or non-cardiac tissues. Upon artificially induced atrial damage, MYBPHL is released into the blood with peak levels reached approximately two hours post-operation and can easily be detected in the plasma. Interestingly, the increase in MYBPHL levels in circulation is correlated with the extent of the atrial damage (endocardial *versus* epicardial) and matches the kinetics of the well-established marker CK-MB. Patients without atrial damage, regardless of whether they are connected to a heart-lung machine or not, never had detectable MYBPHL levels in their plasma which exceeded those measured in healthy individuals. Taken together, our data suggest that MYBPHL may be a useful biomarker for monitoring atrial damage.

## Materials and Methods

### Patients and biopsies

Human tissue biopsies and blood samples were obtained from the cardiovascular biobank (KaBi-DHM) at the German Heart Center Munich. All samples in the KaBi-DHM were obtained with informed consent signed by all participants or their legal guardians. All procedures and sampling were approved by the local ethics committee of the medical faculty at the Technical University of Munich (Project nos. 5943/13 and 223/18S). All study procedures were performed in accordance with relevant guidelines and regulations.

### Assessment of gene expression by qRT-PCR

Frozen human left and right atria, left ventricles, *arteria mammaria interna*, and skeletal muscle samples were minced in 1 mL peqGOLD TriFast^TM^ (Peqlab, Erlangen, Germany) for 30 sec using an Ultraturrax MICCRA D-8 (ART Moderne Labortechnik, Müllheim, Germany). Total RNA was extracted according to the manufacturer’s protocol and reverse-transcribed into cDNA using M-MLV reverse transcriptase (150 U, Invitrogen, Carlsbad, CA), random hexamer primers (375 ng), dNTPs (10 mM each), 10 mM DTT, and 1× first strand buffer in a final volume of 30 µL. Amplification was performed using a QuantStudio 3 (ThermoFisher, Dreieich, Germany) with 5 µM of each primer and Power SYBR green master mix (ThermoFisher). To specifically detect the *MYBPHL* isoforms, we selected primers located in the junction site of the deletion in isoform 2 (Supplementary Table S4). The following conditions were used: 95 °C for 10 min to activate *Taq* polymerase followed by 40 cycles of 95 °C for 15 sec and 60 °C for 1 min. Relative gene expression was normalized to *β-ACTIN* expression as a reference.

### Protein expression in different heart regions

The relative amounts of proteins in different regions of the human heart were quantified using the label-free quantification (LFQ) method from the mass spectrometry data of our previous publication^8^.

### Measurement of MYBPHL in plasma by ELISA

Blood samples were collected in K3-EDTA tubes prior to the operation (Sarstedt, Nürmbrecht, Germany), upon arrival at the intensive care unit (0 h), and then 2, 4, 6, and 24 hours later. Samples were centrifuged for 10 min at 2,000 × *g*. For patients undergoing atrioventricular ablation, blood was drawn prior to the operation and then 6 and 24 hours post-surgery. Plasma was aliquoted (200 µL/aliquot) and frozen immediately at −80 °C. A commercially available ELISA kit (Cat.no. MBS9334464, Mybiosource.com, San Diego, CA) was used to determine the concentration of MYBPHL according to the manufacturer’s instructions. In brief, all reagents were brought to room temperature and 50 µL of undiluted plasma or standards and 100 µL HRP-conjugate were directly pipetted into the ELISA plates and incubated for 1 h at 37 °C. After washing the plates four times, the chromogen solutions were added, and the plates incubated for 15 min at 37 °C in the dark. After the addition of the stop solution, the OD at 450 nm was measured. For each assay, a standard curve was included to calculate the values for individual samples. There was a highly linear correlation up to concentrations of 100 ng/mL, and a lower detection limit of approximately 3 ng/mL (Supplementary Fig. S5). In addition, both intra- and inter-assay variation and the differences between plasma and serum are satisfying (Supplementary Figs S6, S7 and S8).

### Statistical analysis

Relative gene expression of candidate genes was calculated with the relative expression software tool (REST^©^)^25^ using *β-ACTIN* as a reference. For the expression of *MYBPHL*, *MYBPHL* isoform 1 and 2, *MYBPH*, *MYBPC3*, and *FHL2* in tissues, standard curves were established to determine the arbitrary units of gene expression with *β-ACTIN* as a reference. The two-sided *t*-test was used to determine significant differences. To assess the significance between plasma concentrations at different time points, the one-sided *t*-test was applied, except for the difference between healthy controls and MAZE patients (Fig. 4A), where a two-sided *t*-test was used. In all cases, a p-value of <0.05 was considered to be significant.

## Supplementary information

Dataset 1
