# Supplementary material for: Myosin binding protein H-like (MYBPHL): a promising biomarker to predict atrial damage
Source: Sci Rep. 2019 Jul 10;9:9986. doi: 10.1038/s41598-019-46123-w (PMC6620353; doi:10.1038/s41598-019-46123-w)

Myosin binding protein H-like (MYBPHL): a promising biomarker to predict atrial damage

Harald Lahm, Martina Dreßen, Nicole Beck, Stefanie Doppler, Marcus-André Deutsch, Matsushima Shunsuke, Irina Neb, Karl Christian König, Konstantinos Sideris, Stefanie Voss, Lena Eschenbach, Nazan Puluca, Isabel Deisenhofer, Sophia Doll, Stefan Holdenrieder, Matthias Mann, Rüdiger Lange, Markus Krane

Figure S1. Relative protein expression of candidate genes in 16 human heart regions.

Figure S2. CK-MB values in plasma of patients with atrial fibrillation.

Figure S3. Troponin T values in plasma of patients with atrial fibrillation.

Figure S4. MYBPHL in patients with atrioventricular node ablation.

Figure S5. Sensitivity of the MYBPHL ELISA.

Figure S6. Intra-assay variation of samples.

Figure S7. Inter-assay variation of samples.

Figure S8. Comparison of MYBPHL values in plasma and serum samples.

Table S1. MAZE patients.

Table S2. AVR and TAVI patients.

Table S3. Patients with atrioventricular node ablation.

Table S4. Primers used for qRT-PCR analysis.

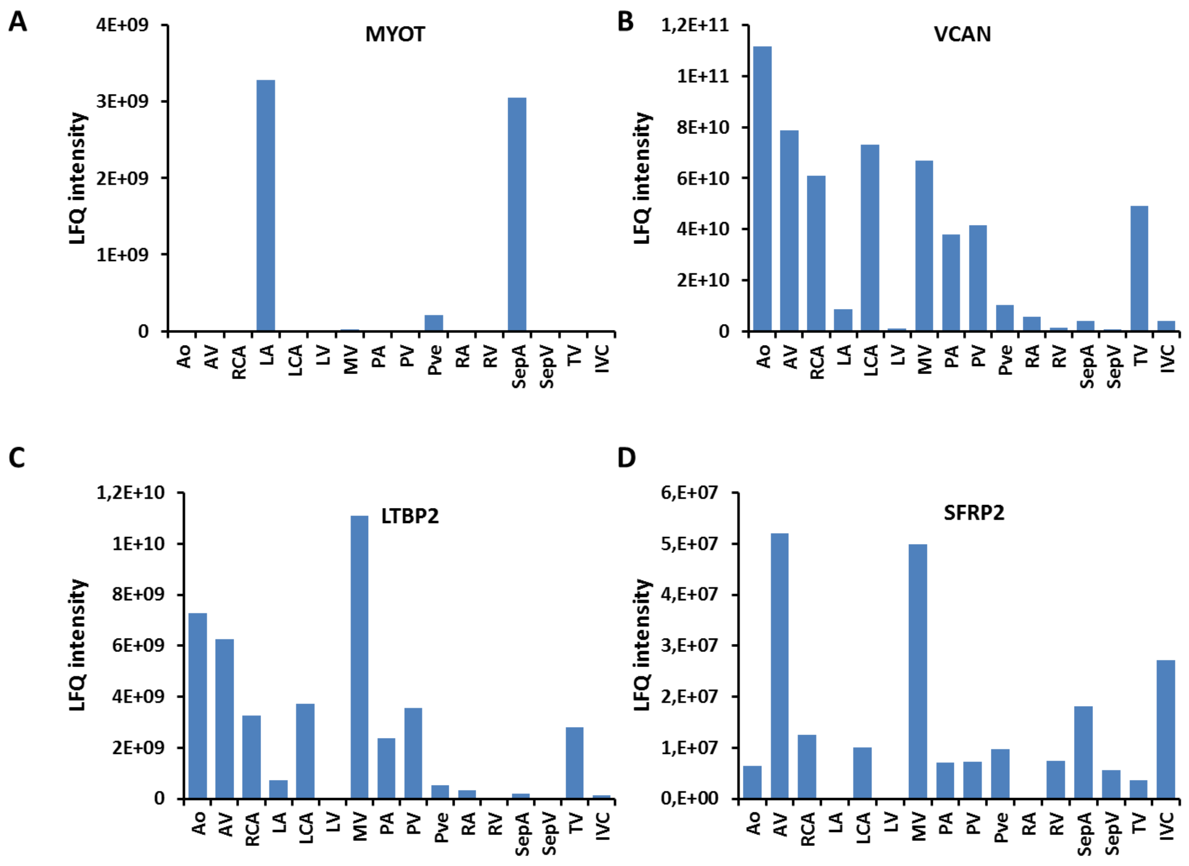

Figure S1. Relative protein amount of candidate genes in 16 regions of the human heart. Protein amounts are represented as label-free quantification intensity.

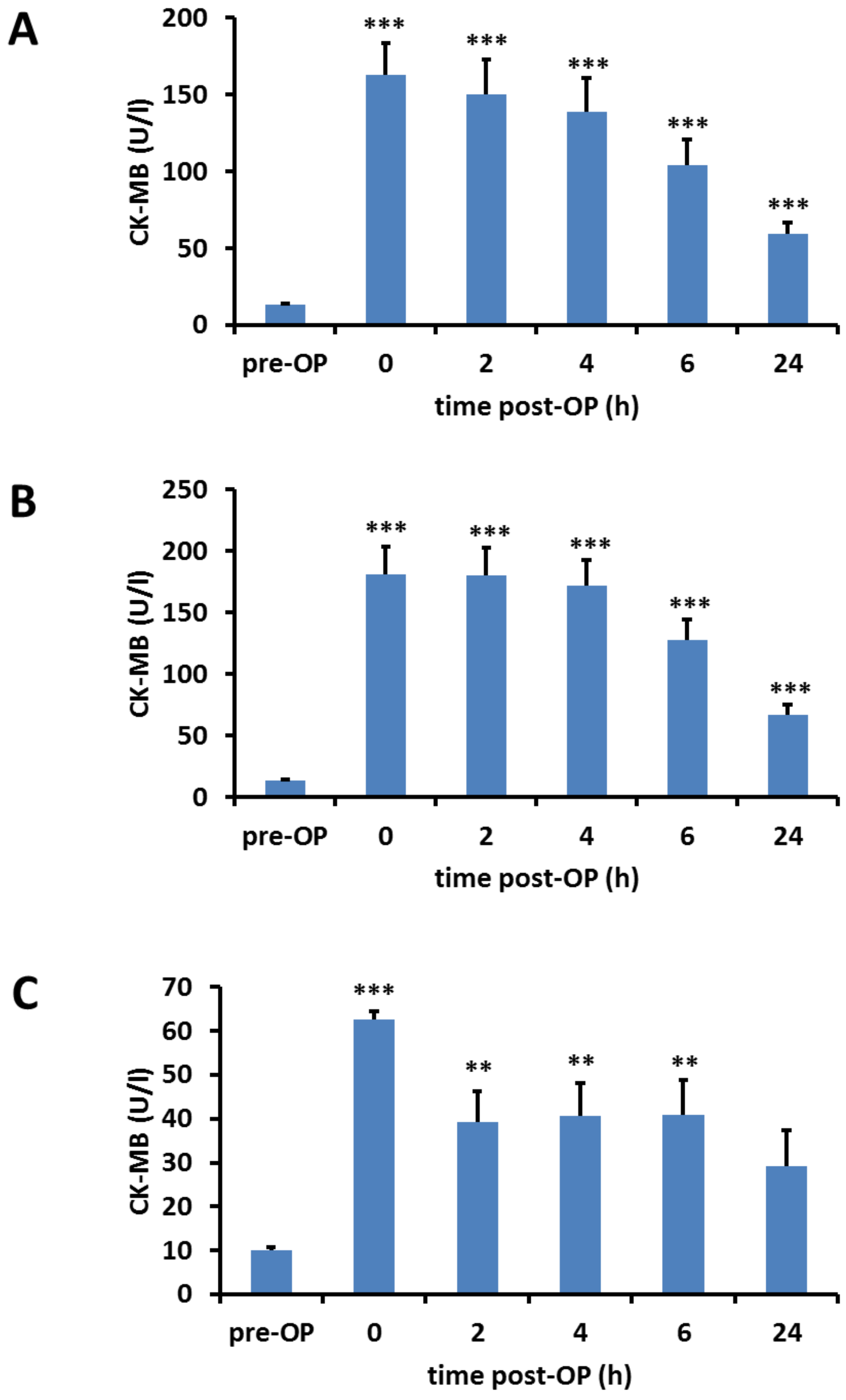

Figure S2. CK-MB values in plasma of patients with atrial fibrillation. **A:** Time course of CK-MB in patients with atrial fibrillation receiving cryo-ablation (n = 17). **B, C:** Time course of CK-MB expression in atrial patients with endo- (**B**) (n = 12) or epicardial (**C**) (n = 5) cryo-ablation. Values represent the mean ± SE. **: $p < 0.01$, *** $p < 0.001$.

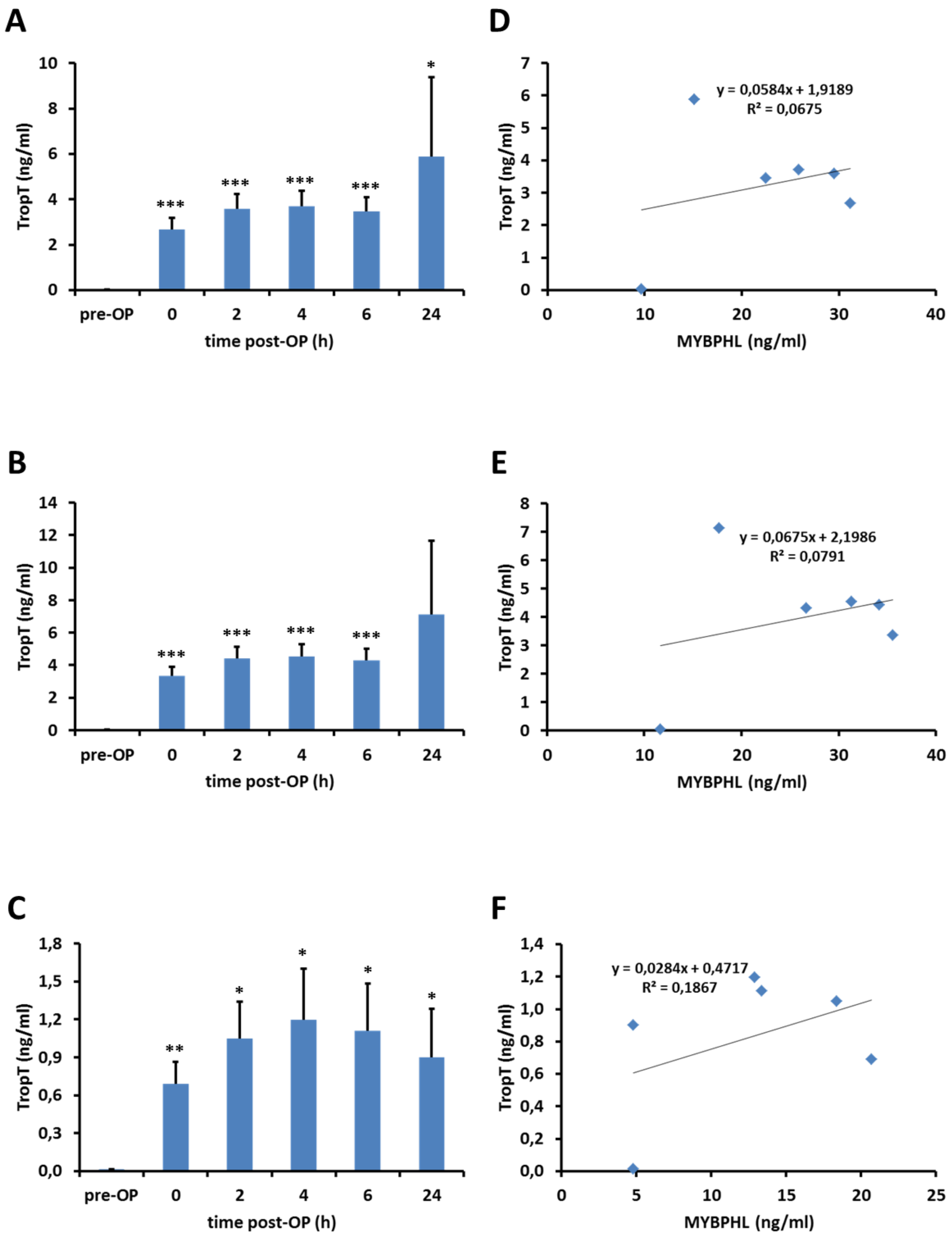

Figure S3. Troponin T values in plasma of patients with atrial fibrillation. **A:** Time course of troponin T in patients with atrial fibrillation receiving cryo-ablation (*n* = 17). **B, C:** Time course of troponin T expression in atrial patients with endo- (**B**) (*n* = 12) or epicardial (**C**) (*n* = 5) cryo-ablation. **D - F:** Correlation between MYBPHL and troponin T. Values represent the mean ± SE. *: $p < 0.05$, **: $p < 0.01$, *** $p < 0.001$.

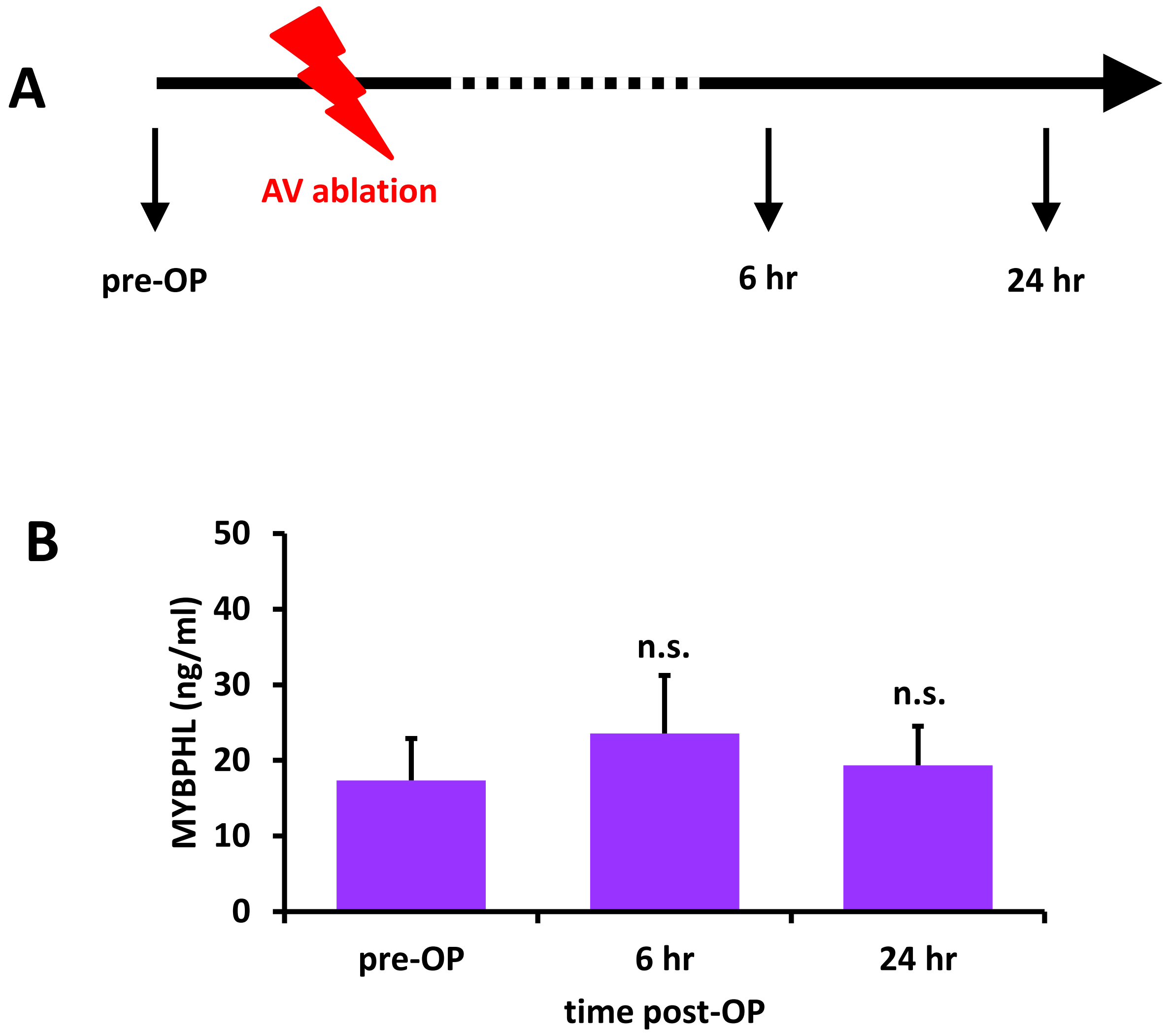

Figure S4: MYBPHL in patients with atrioventricular node ablation.

A: Scheme of serum sampling**. B:** Time course of MYBPHL in patients with atrial node ablation (n = 6). Values are presented as the mean ± SEM. n.s.: not significant.

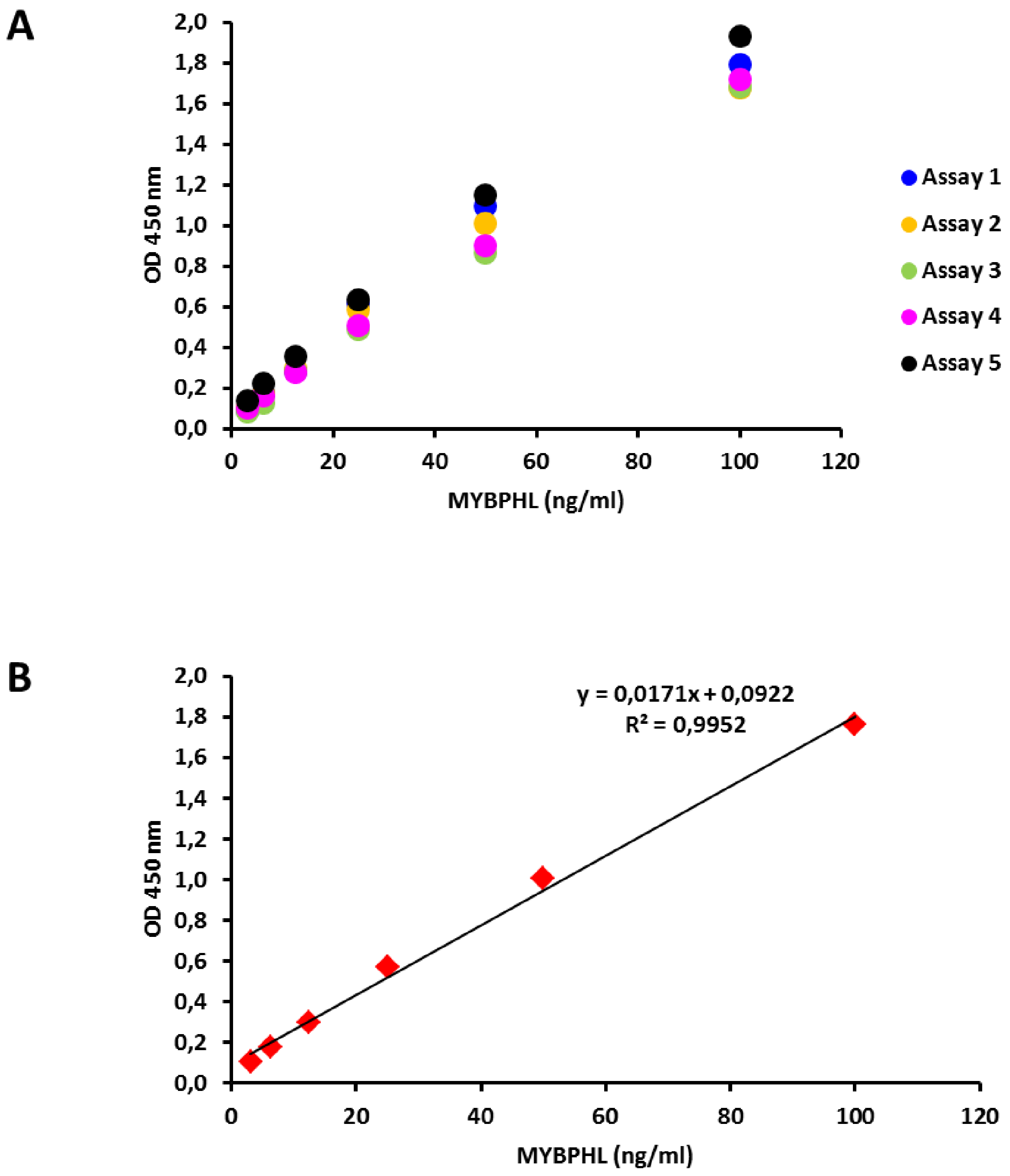

Figure S5. Sensitivity of the MYBPHL ELISA. A: OD450 nm values of graded MYBPHL concentrations measured in five independent assays. **B:** Cumulative standard curve obtained by five independent assays.

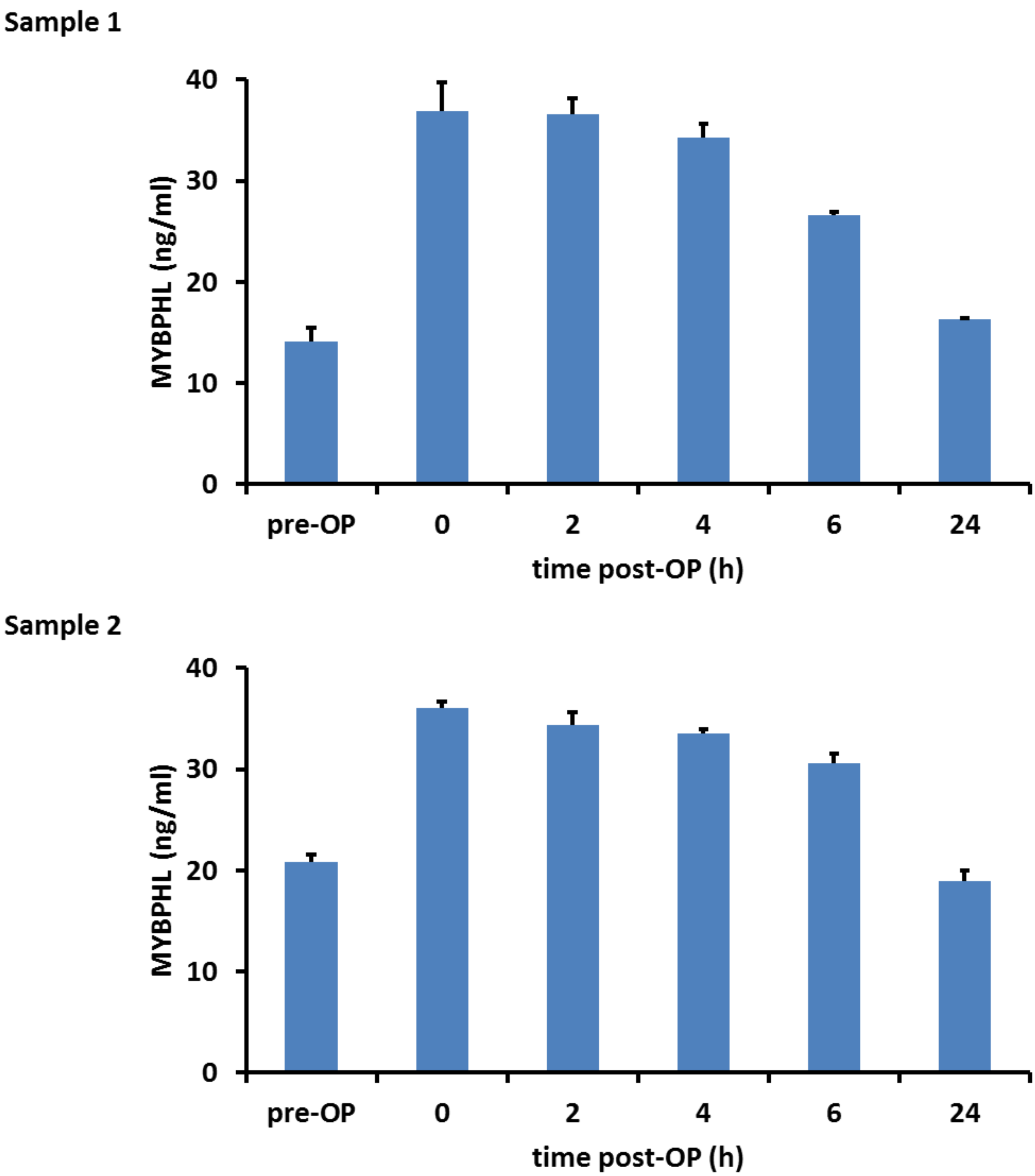

Figure S6. Intra-assay variation of samples. Representative examples of two different patients where plasma samples were applied on the same microtiter plate. Values represent the mean ± SE.

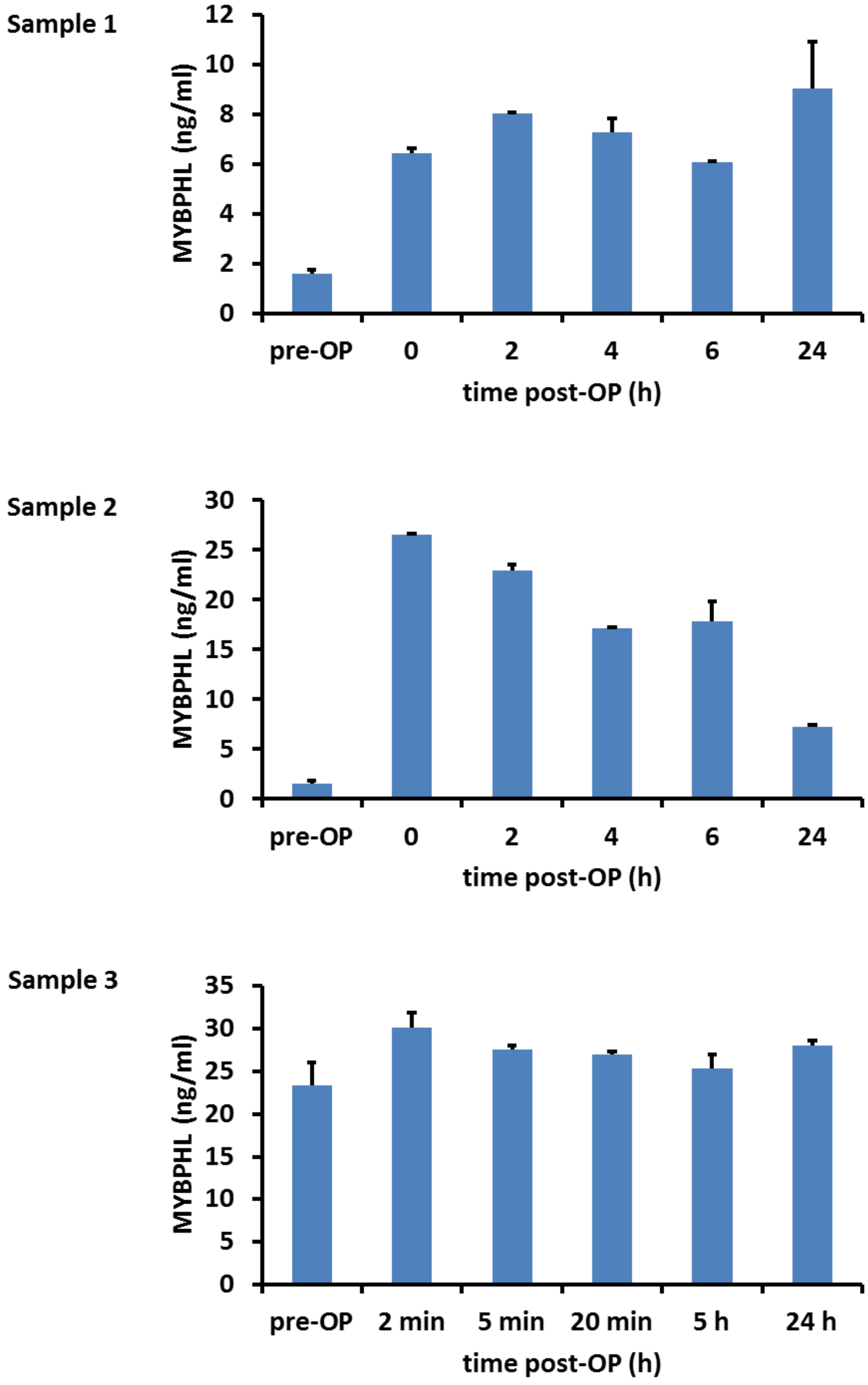

Figure S7. Inter-assay variation of samples. Representative examples of three different patients where plasma samples were applied on two different microtiter plates. Values represent the mean ± SE.

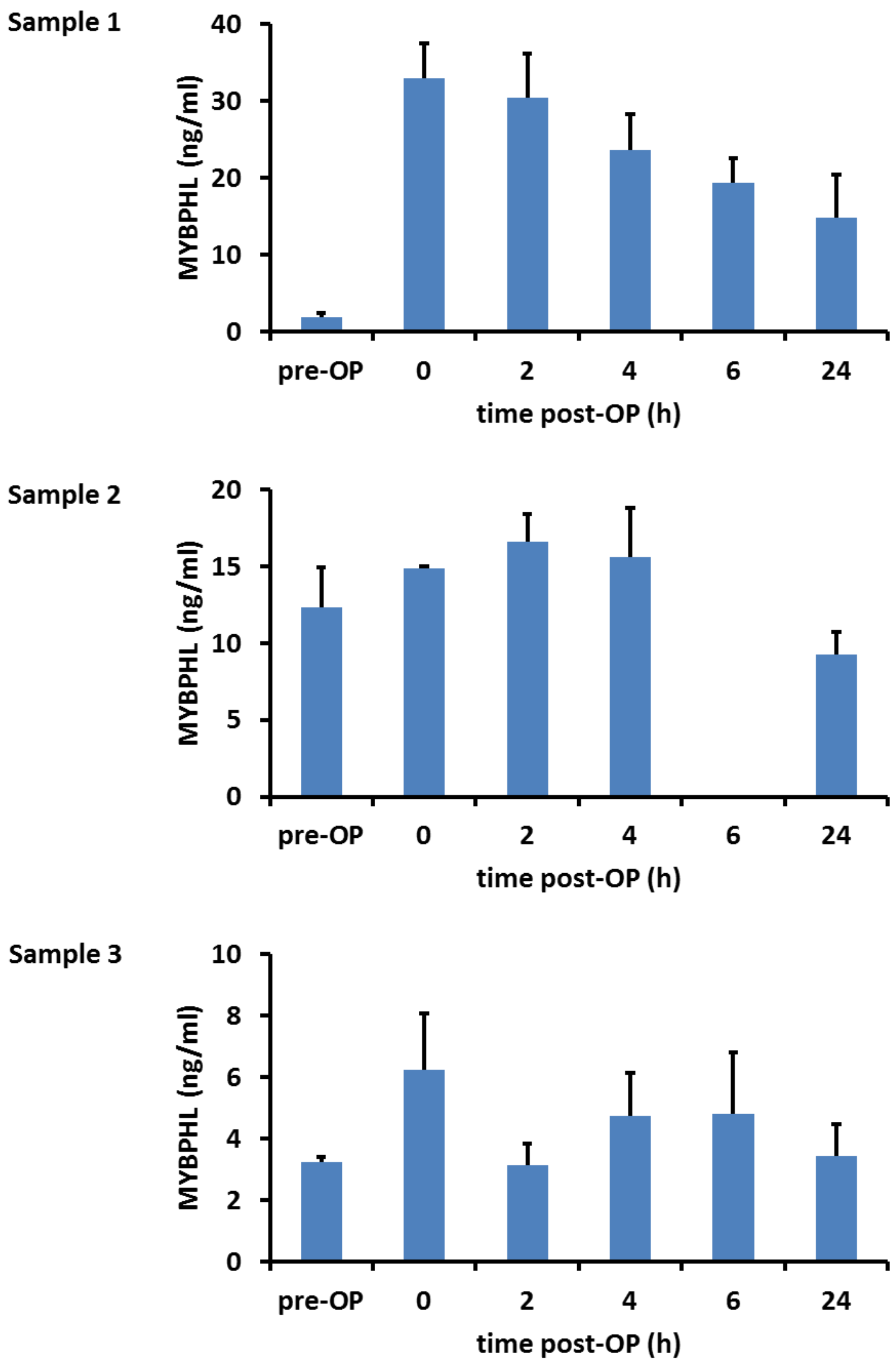

Figure S8. Comparison of MYBPHL values in plasma and serum samples. Representative examples of three different patients where plasma and serum samples were analyzed for their MYBPHL concentration. Values represent the mean ± SE.

Supplementary Table S1. MAZE Patients.

patient	age	sex (m/f)	HLM* (min)	ablation	clinical procedure**
#1	61	female	98	endocardial	MVR
#2	64	male	129	endocardial	MVR
#3	75	female	111	endocardial	MVR + TVR
#4	67	male	110	endocardial	MVR + TVR
#5	69	female	78	endocardial	MVR
#6	77	female	87	endocardial	MVR + TVR
#7	66	male	97	endocardial	AVR
#8	74	male		endocardial	AVR + MVR
#9	69	female	107	endocardial	TVR
#10	65	female	73	endocardial	MVR + TVR
#11	78	male	71	endocardial	MVR
#12	52	female	90	endocardial	MVR
	68.1±2.1		95.6±5.0		
#13	59	male	37	epicardial	HOCM
#14	77	male	118	epicardial	AVR + ACVB
#15	81	female	59	epicardial	ACVB
#16	78	male	80	epicardial	AVR
#17	80	female	101	epicardial	ACVB
	75.0±3.6		79.0±12.9		

*: heart-lung machine, **: AVR: aortic valve replacement, MVR: mitral valve replacement, TVR: tricuspid valve replacement, ACVB: aortocoronary venous bypass, HOCM: hypertrophic obstructive cardiomyopathy.

Supplementary Table S2. AVR and TAVI patients.

patient	age	sex (m/f)	HLM* (min)	ablation	clinical procedure**
aortic valve replacement					
#1	66	female	59	none	AVR
#2	63	male	50	none	AVR
#3	75	female	52	none	AVR
#4	70	female	73	none	AVR
#5	73	female	64	none	AVR
	69.4±2.0		59.6±3.7		
transcatheter aortic valve implantation					
#1	69	male	none	none	transfemoral implantation
#2	76	male	none	none	transfemoral implantation
#3	78	male	none	none	transfemoral implantation
#4	90	male	none	none	transfemoral implantation
#5	64	male	none	none	transfemoral implantation
	75.4±4.0				
Controls	35.1±2.9	4 / 7			

*: heart-lung machine, **: AVR: aortic valve replacement.

Supplementary Table S3. Patients with atrioventricular node ablation.

patient	age	sex (m/f)	HLM* (min)	ablation	clinical procedure
transcatheter aortic valve implantation					
#1	90	female	none	radiofrequency	ablation of atrioventricular node
#2	81	male	none	radiofrequency	ablation of atrioventricular node
#3	74	female	none	radiofrequency	ablation of atrioventricular node
#4	36	female	none	radiofrequency	ablation of atrioventricular node
#5	75	female	none	radiofrequency	ablation of atrioventricular node
#6	55	male	none	radiofrequency	ablation of atrioventricular node
	68.5±7.3	2 / 4			

*: heart-lung machine.

Supplementary Table S4. Primers used for qRT-PCR analysis

primer	sequence	length of amplicon
hu β-actin_F382	5’ CCA ACC GCG AGA AGA TGA 3’	
hu β-actin_R478	5’ CCA GAG GCG TAC AGG GAT AG 3’	96 bp
hu FHL2_F1076	5‘ GTG CAC CAA CCC CAT CAG 3‘	
hu FHL2_R1184	5‘ CAC CAG TGA GAG GGA GCA CT 3‘	108 bp
hu LTBP_F4097	5‘ AGG GCT CCT TCA ACT GTC TAT G 3‘	
hu LTBP_R4187	5‘ CCA TAG TCC TCA CAC TCG TCA A 3‘	90 bp
hu MYBPC3_F320	5‘ AAG TTC GAC CTC AAG GTC ATA GA 3‘	
hu MYBPC3_R412	5‘ TTC TCC AGG GGC TCC AGT 3‘	92 bp
hu MYBPH_F548	5‘ CCA CAT CCG AGA GAA CAT TG 3‘	
hu MYBPH_R623	5‘ CAC CTG GCG GAT GTA GGT 3‘	75 bP
hu MYBPHL_F249	5‘ GTT GGG GAC ACA GTG AAC CT 3‘	
hu MYBPHL_R319	5‘ TCA TGT GTC CAG ATG GCT TG 3‘	70 bp
hu MYBPHL_t1_F405	5‘ GAC TCA GGT CGC TAC CAA CT 3‘	
hu MYBPHL_t1_R485	5‘ CTC AAT CAC CAG GAT GTC AAT G 3‘	80 bp
hu MYBPHL_t2_F401	5‘ TGC TGA CTC AGA GAG GCC A 3‘	
hu MYBPHL_t2_R586	5‘ GTG CGG TGA TAG TGC TCC AG 3‘	185 bp
hu MYOT_F1478	5‘ AAT TCA ACA CTG ACC GAA TAA GC 3‘	
hu MYOT_R1569	5’ CCA CCC AGC ATC TTT CTT GT 3’	91 bp
hu PAM_F960	5‘ TTG CTG GCA TGT ACC CTA TGA 3‘	
hu PAM_R1038	5‘ CAT GAA ATG TCA GAA TTC ACC AC 3‘	78 bp
hu SFRP2_F555	5‘ GCT GGA GCA CGA GAC CAT 3‘	
hu SFRP2_R629	5‘ TGG CAG TTC TTG TTG AGC A 3‘	74 bp
hu VCAN_F9694	5‘ GCA CCT GTG TGC CAG GAT 3‘	
hu VCAN_R9763	5‘ CAG GGA TTA GAG TGA CAT TCA TCA 3‘	69 bp

Myosin binding protein H-like (MYBPHL): a promising biomarker to predict atrial damage

Harald Lahm, Martina Dreßen, Nicole Beck, Stefanie Doppler, Marcus-André Deutsch, Matsushima Shunsuke, Irina Neb, Karl Christian König, Konstantinos Sideris, Stefanie Voss, Lena Eschenbach, Nazan Puluca, Isabel Deisenhofer, Sophia Doll, Stefan Holdenrieder, Matthias Mann, Rüdiger Lange, Markus Krane

Supplementary information:
Complete gels of qRT-PCR analyses

Left atrium: MYBPHL

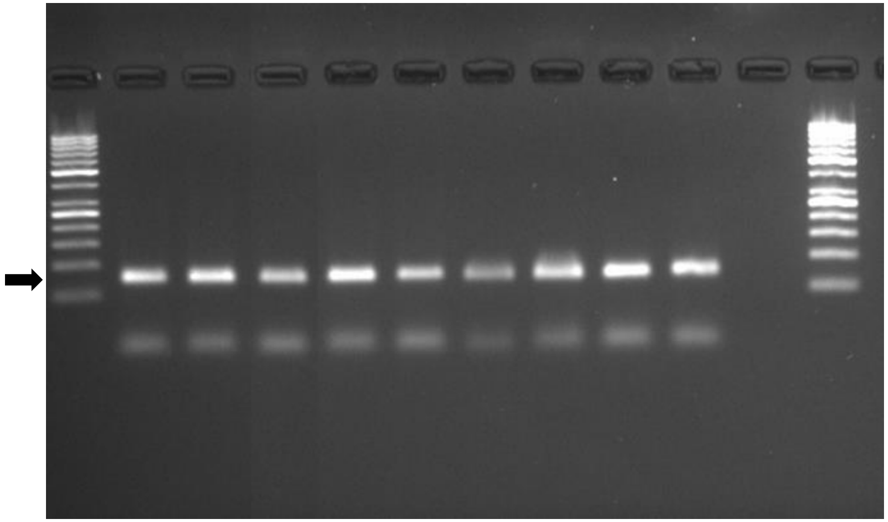

Left atrium: MYBPHL (isoform 1)

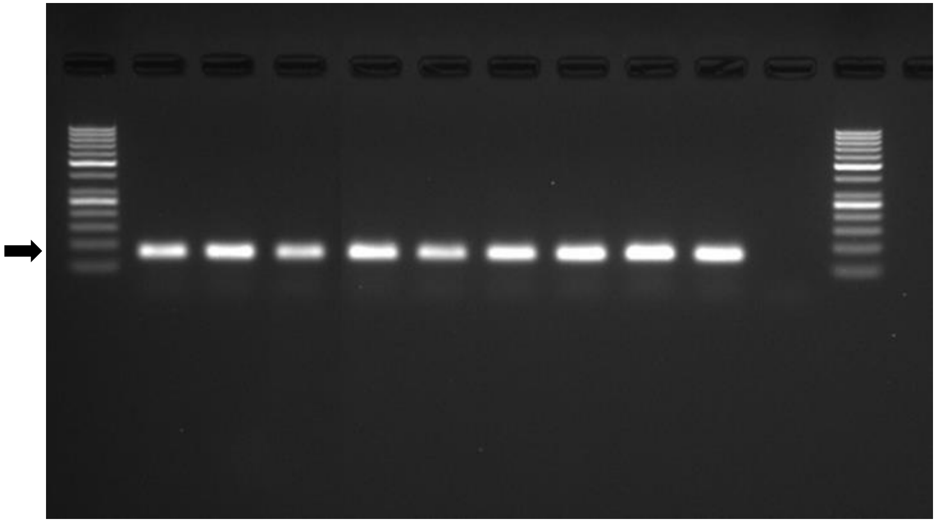

Left atrium: MYBPHL (isoform 2)

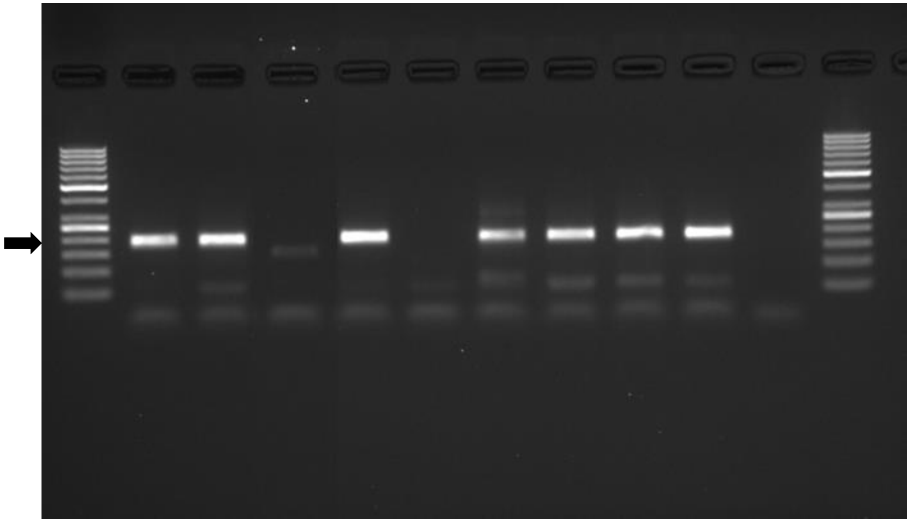

Left atrium: MYBPH

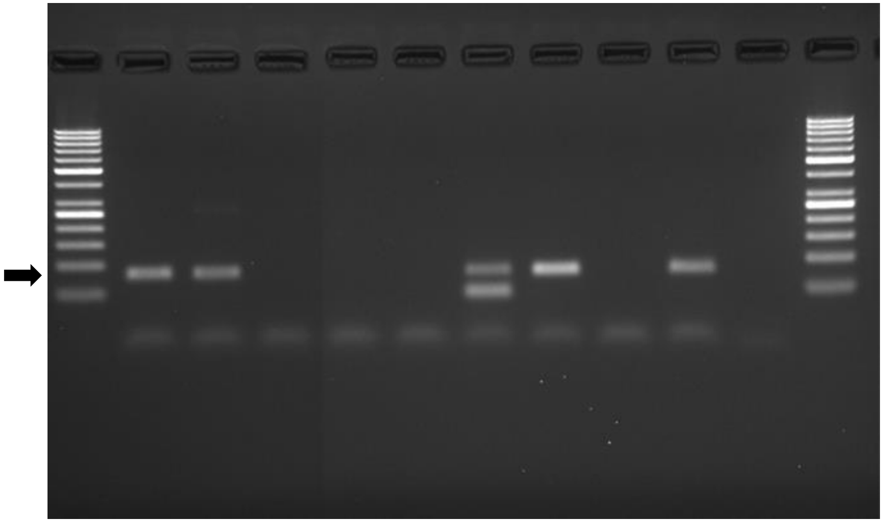

Left atrium: MYBPC3

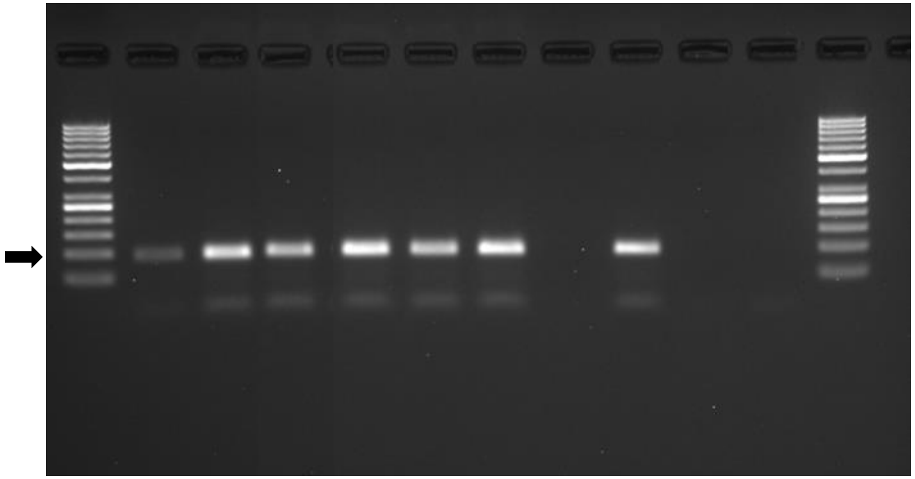

Left atrium: FHL2

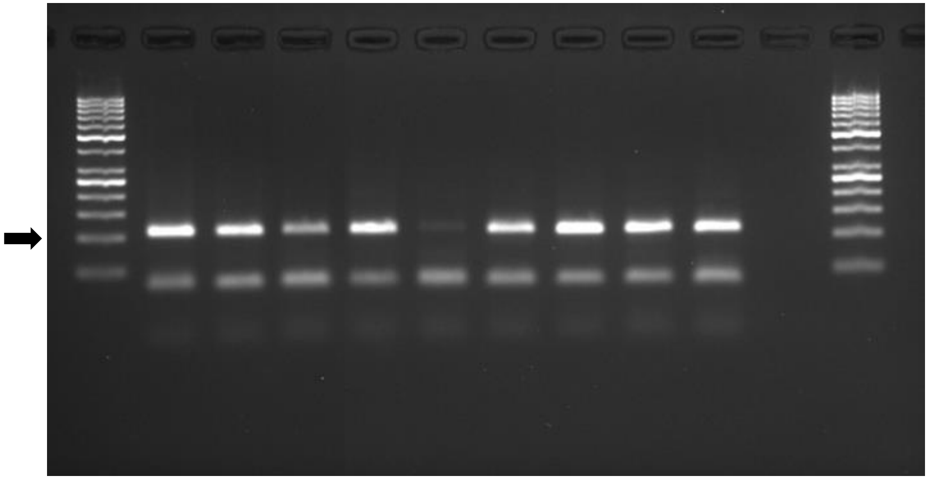

Left atrium: beta-actin

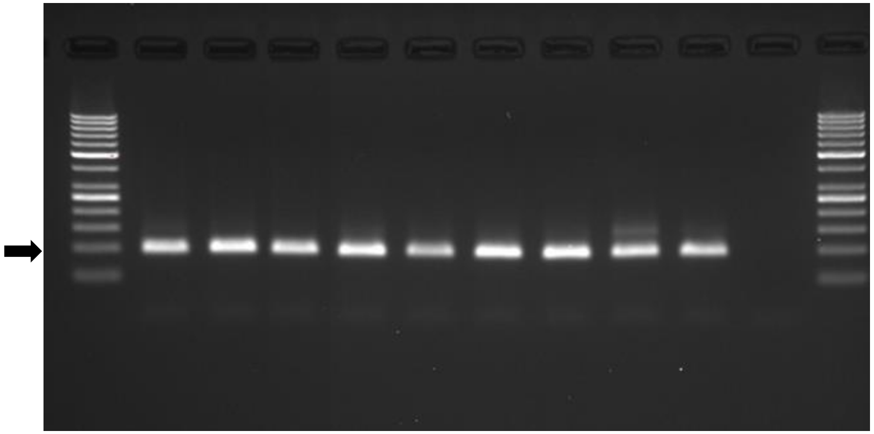

Left ventricle: MYBPHL

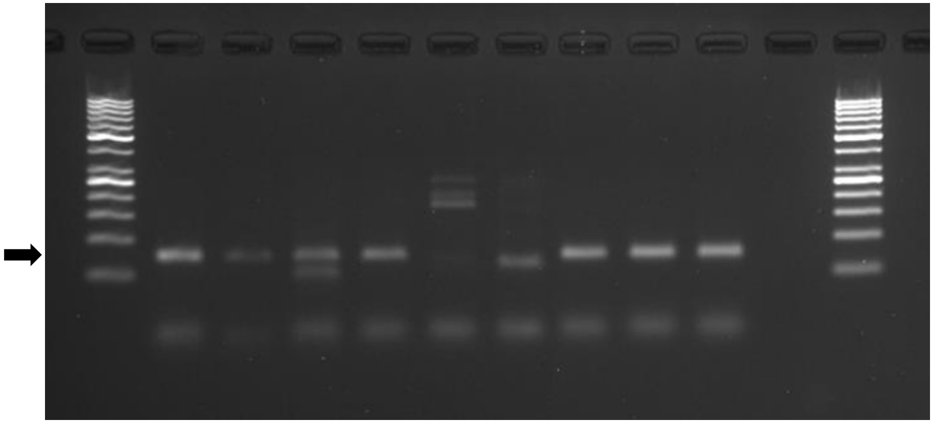

Left ventricle: MYBPHL (isoform 1)

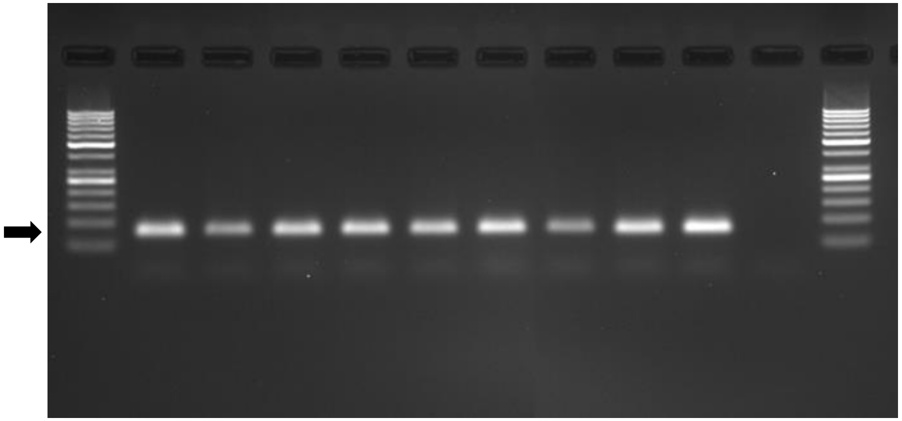

Left ventricle: MYBPHL (isoform 2)

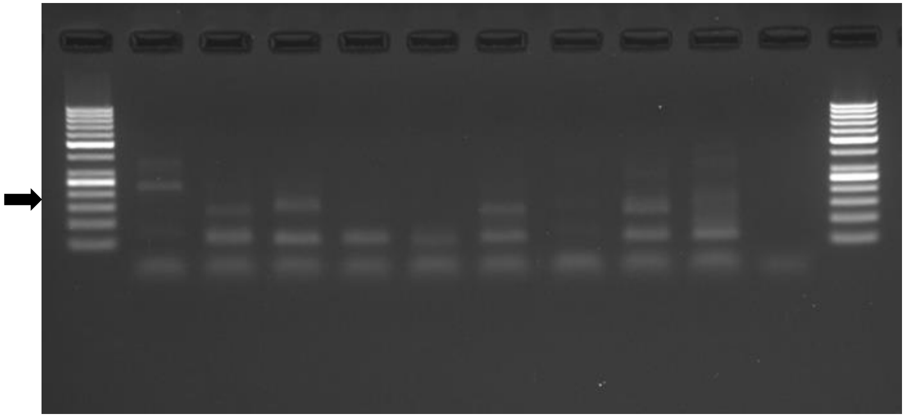

Left ventricle: MYBPH

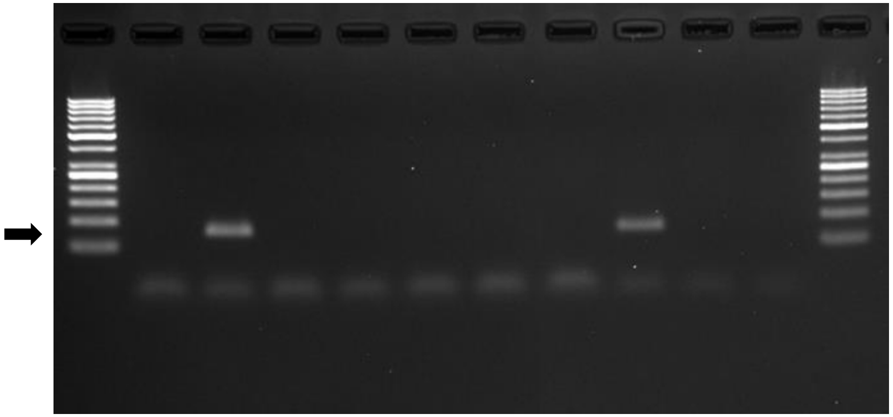

Left ventricle: MYBPC3

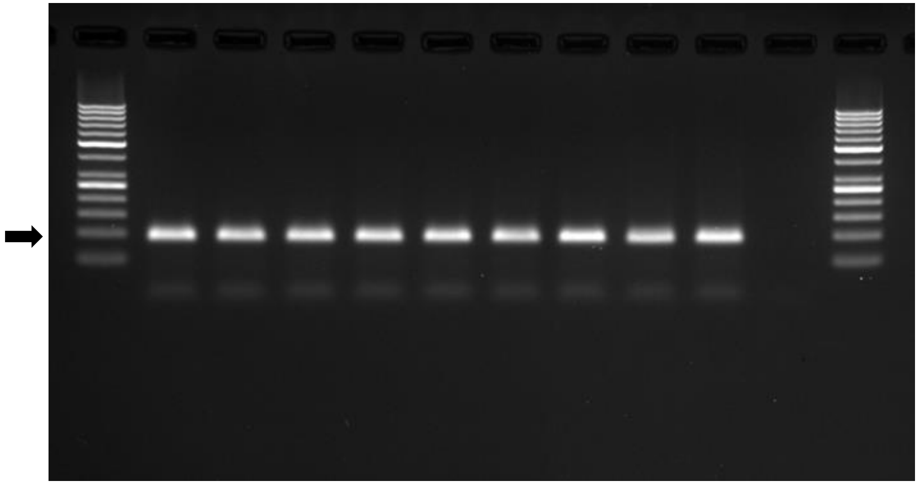

Left ventricle: FHL2

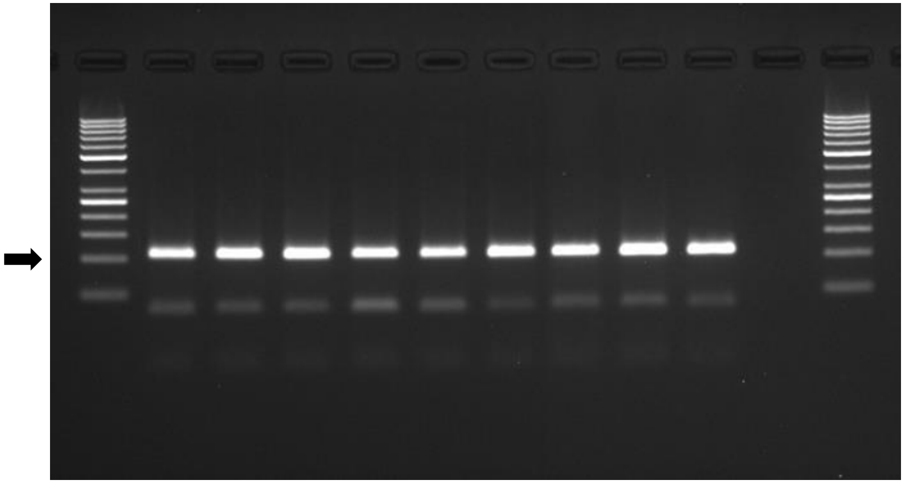

Left ventricle: beta-actin

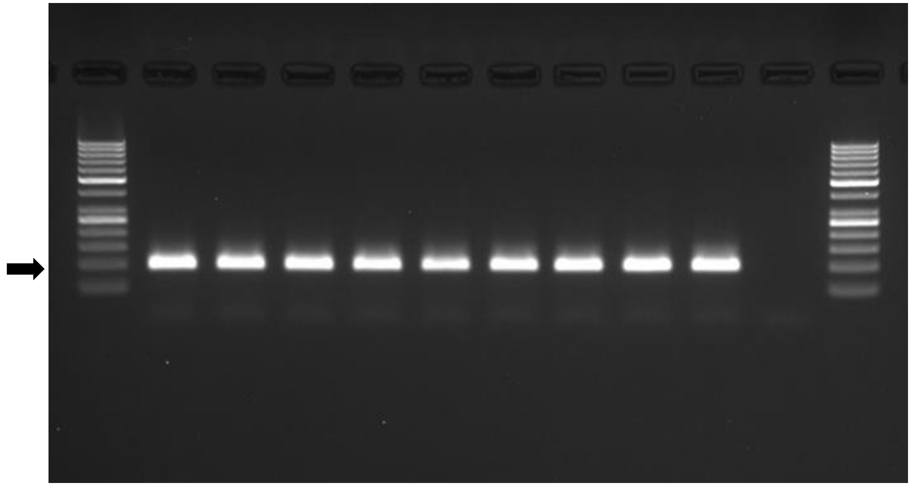

arteria mammaria interna: MYBPHL

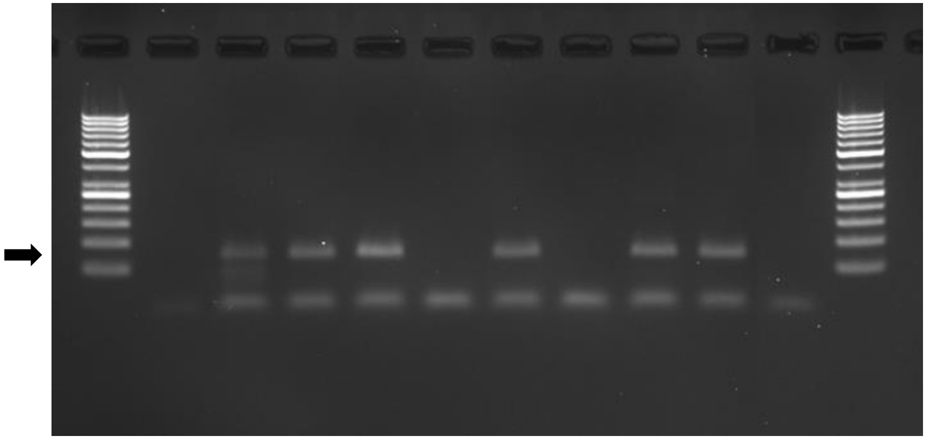

arteria mammaria interna: MYBPHL (isoform 1)

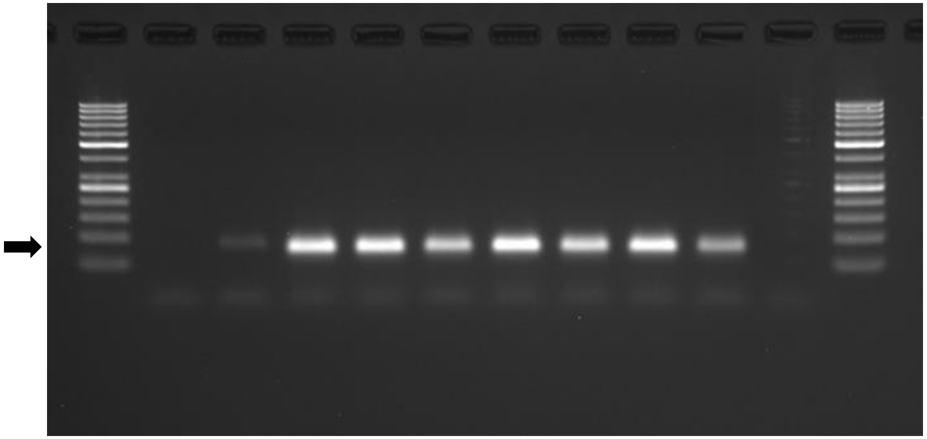

arteria mammaria interna: MYBPHL (isoform 2)

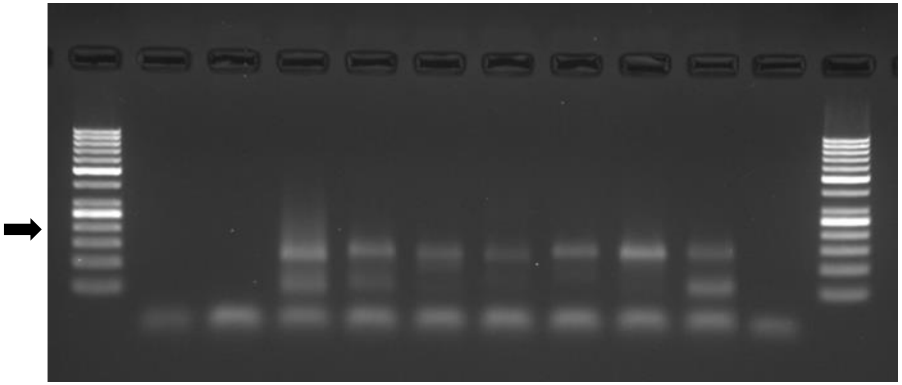

arteria mammaria interna: MYBPH

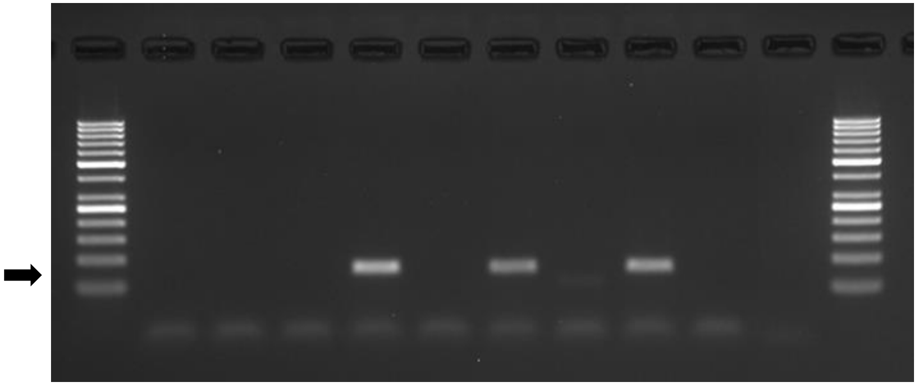

arteria mammaria interna: MYBPC3

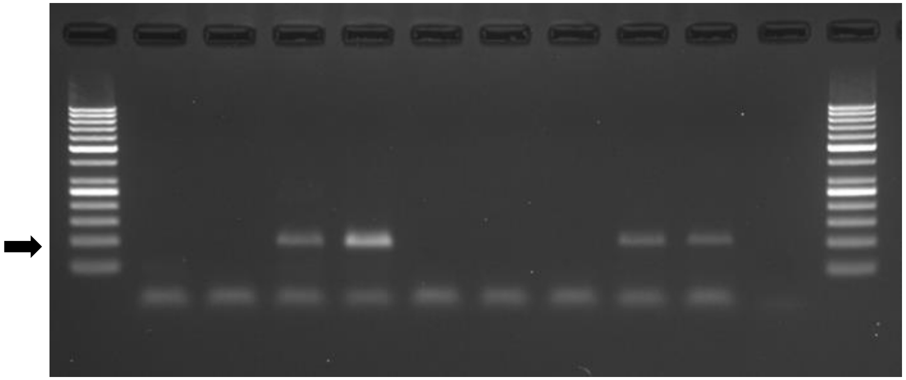

arteria mammaria interna: FHL2

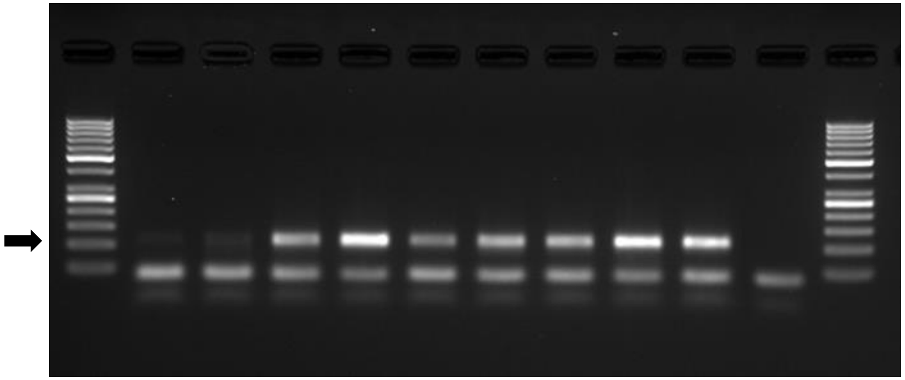

arteria mammaria interna: beta-actin

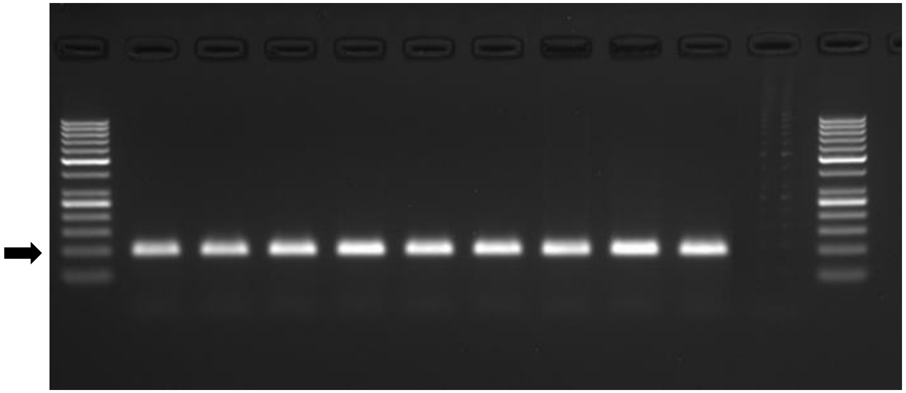

skeletal muscle: MYBPHL

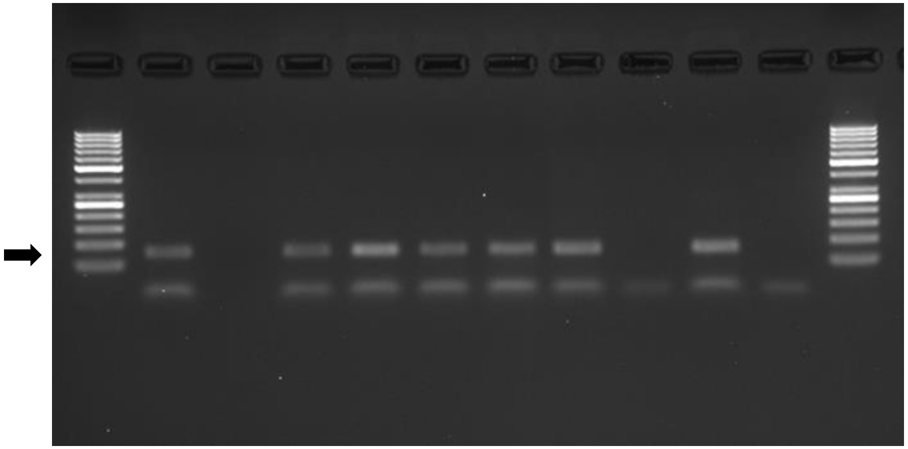

skeletal muscle: MYBPHL (isoform 1)

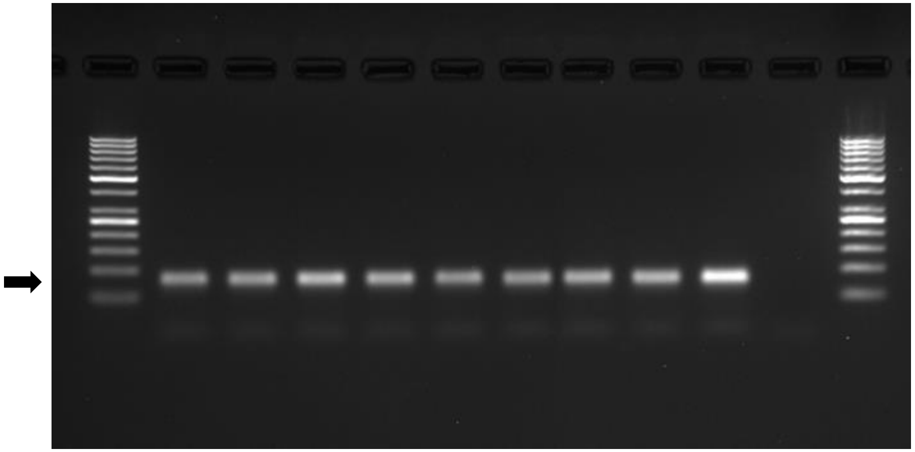

skeletal muscle: MYBPHL (isoform 2)

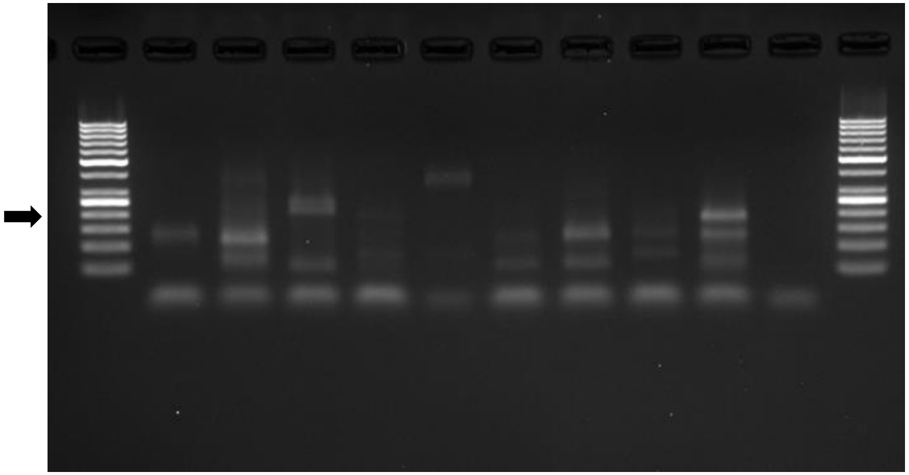

skeletal muscle: MYBPH

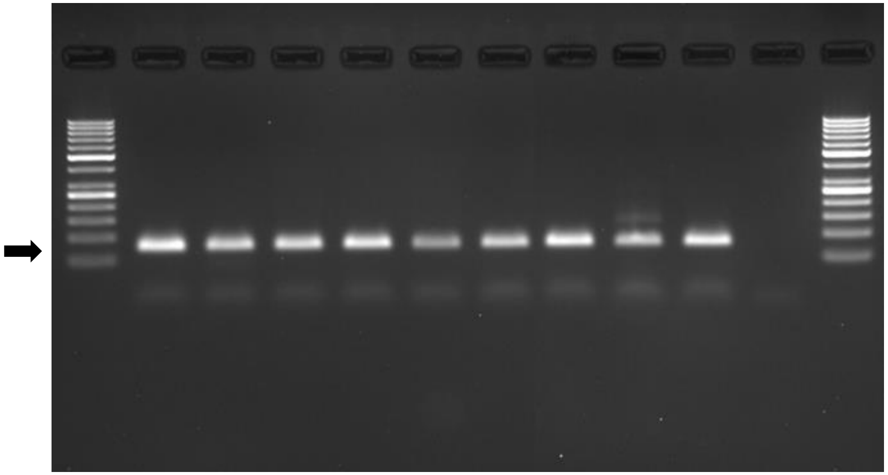

skeletal muscle: MYBPC3

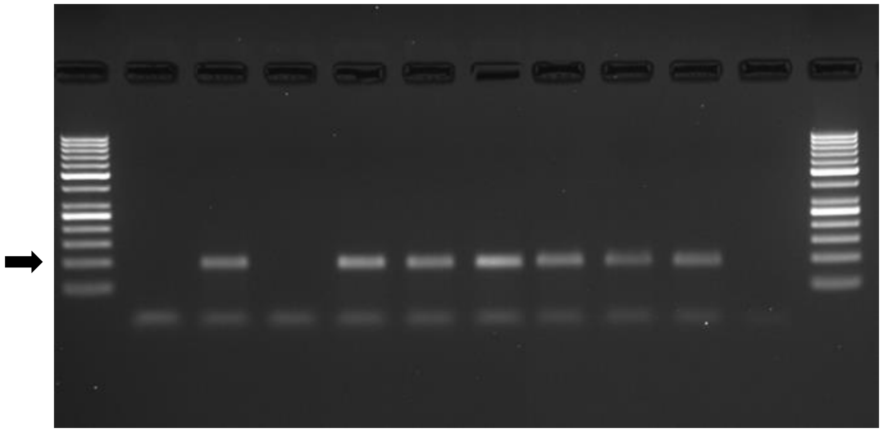

skeletal muscle: FHL2

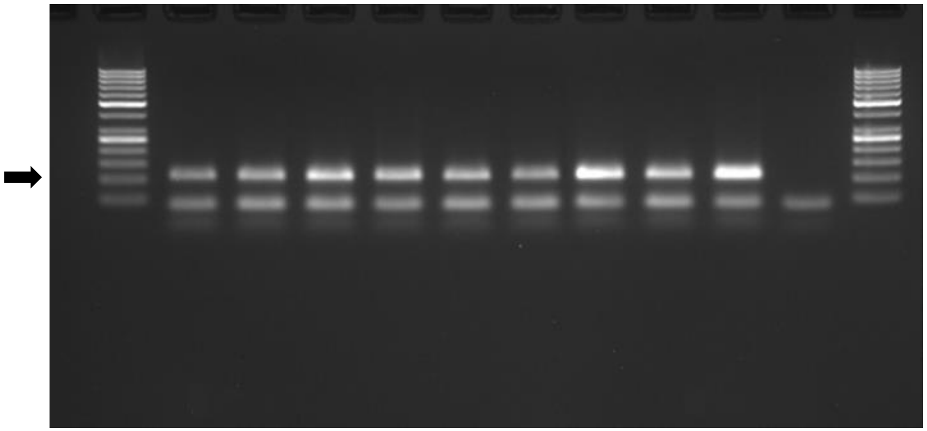

skeletal muscle: beta-actin

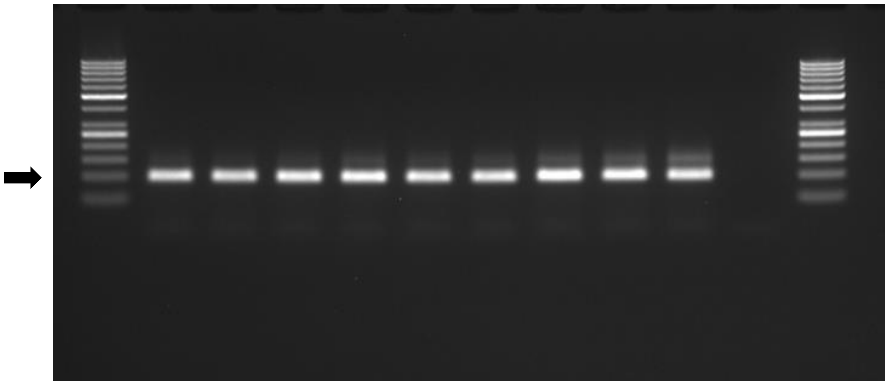

Supplement: Supplementary file 1 — Dataset 1 [file 41598_2019_46123_MOESM1_ESM.pdf]
